# The Inverted U: Exploring the Interconnectedness of Movement and Cognitive Function Across the Lifespan

**DOI:** 10.1002/wcs.70020

**Published:** 2026-01-21

**Authors:** Gerry Leisman, Rahela Alfasi, Oded Meiron, Amedeo D'Angiulli

**Affiliations:** ^1^ Movement & Cognition Laboratory, Department of Physical Therapy University of Haifa Haifa Israel; ^2^ Resonance Therapeutics Laboratory, Department of Neurology University of the Medical Sciences of Havana Havana Cuba; ^3^ Faculty of Education, Bar‐Ilan University Ramat Gan Israel; ^4^ Clinical Research Center for Brain Sciences Herzog Medical Center Jerusalem Israel; ^5^ NICER Laboratory, Department of Neuroscience Carleton University Ottawa Canada; ^6^ Children's Hospital of Eastern Ontario Research Institute Ottawa Canada

**Keywords:** adolescence, aging, childhood, cognition, fetal cognition, human development, infancy, inverted U, learning, movement

## Abstract

The “inverted U” relationship between movement and cognition throughout the human lifespan highlights the intricate interplay between physical activity and cognitive function. This relationship posits that an optimal level of physical activity maximizes cognitive function, while insufficient activity can lead to suboptimal cognitive outcomes. This phenomenon is observed from fetal development to old age, emphasizing the importance of maintaining a balance in physical activity for overall well‐being. During fetal development, maternal physical activity positively influences fetal brain growth, laying the foundation for future cognitive and physical functioning. As the child develops, regular physical activity supports improvements in key cognitive functions such as attention, memory, and executive function abilities essential for learning and academic success. In adulthood, maintaining an active lifestyle continues to play a central role in preserving cognitive abilities and reducing the risk of neurodegenerative diseases such as Alzheimer's. The inverted U model suggests that optimal cognitive functioning is achieved at moderate levels of physical activity, while too little activity can be detrimental. In older adulthood, regular physical activity is vital for maintaining cognitive function, slowing cognitive decline, and improving quality of life. In summary, understanding the balance between physical activity and cognition across the lifespan is essential for promoting cognitive resilience and sustained well‐being.

This article is categorized under:

Cognitive Biology > Cognitive Development
Psychology > Development and Aging
Psychology > Learning

## Graphical abstract

The inverted U in Movement and Cognition

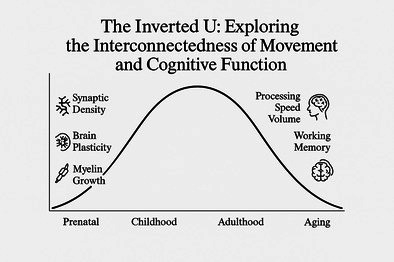

## 1 Introduction

The concept of the “inverted U” relationship between movement and cognition throughout the human lifespan underscores the intricate interplay between physical activity and cognitive function. This relationship suggests that an optimal level of physical activity maximizes cognitive function, while insufficient activity may lead to suboptimal cognitive outcomes. This phenomenon is observed across various stages of life, from fetal development to old age, highlighting the importance of maintaining an appropriate level of physical activity to promote overall well‐being.

During fetal development, the foundation for future cognitive and physical health is established (Leisman et al. 2024). Research indicates that maternal physical activity can positively influence fetal brain development (Gomes da Silva and Arida 2015), potentially leading to better cognitive outcomes in childhood and beyond. This early influence underscores the importance of a healthy lifestyle during pregnancy, not only for the physical health of the mother but also for the child's cognitive development.

As children grow, physical activity continues to play a crucial role in cognitive development. Engaging in regular physical activity has been shown to enhance various aspects of cognitive function (Young et al. 2015), including attention (De Greeff et al. 2018; Anguera et al. 2022), memory (Düzel et al. 2018; Zhidong et al. 2021), and executive function (EF). These benefits are partly mediated by neurobiological mechanisms such as increased synaptic plasticity, enhanced myelination of frontoparietal networks, and elevated neurogenesis, especially in brain regions supporting cognitive control and decision‐making (Düzel et al. 2018; Li et al. 2024). These mechanisms support the development of cognitive flexibility, working memory, and inhibitory control, all of which are crucial during the school years, when academic performance and learning are closely linked to cognitive abilities. Children who are more physically active tend to perform better academically (Ferreira Vorkapic et al. 2021), suggesting that schools should prioritize physical education and active play to support cognitive development.

The connection between exercise and mental function is still important in maturity. A lower risk of cognitive decline and neurodegenerative disorders like Alzheimer's disease is linked to regular physical activity. The mechanisms underlying this protective effect are thought to include improved cardiovascular health, increased neurogenesis, and enhanced synaptic plasticity. According to Li et al. (2024), adults who engage in moderate to vigorous physical activity exhibit better cognitive function compared to their sedentary peers, emphasizing the importance of maintaining an active lifestyle throughout adulthood.

**FIGURE 1:**
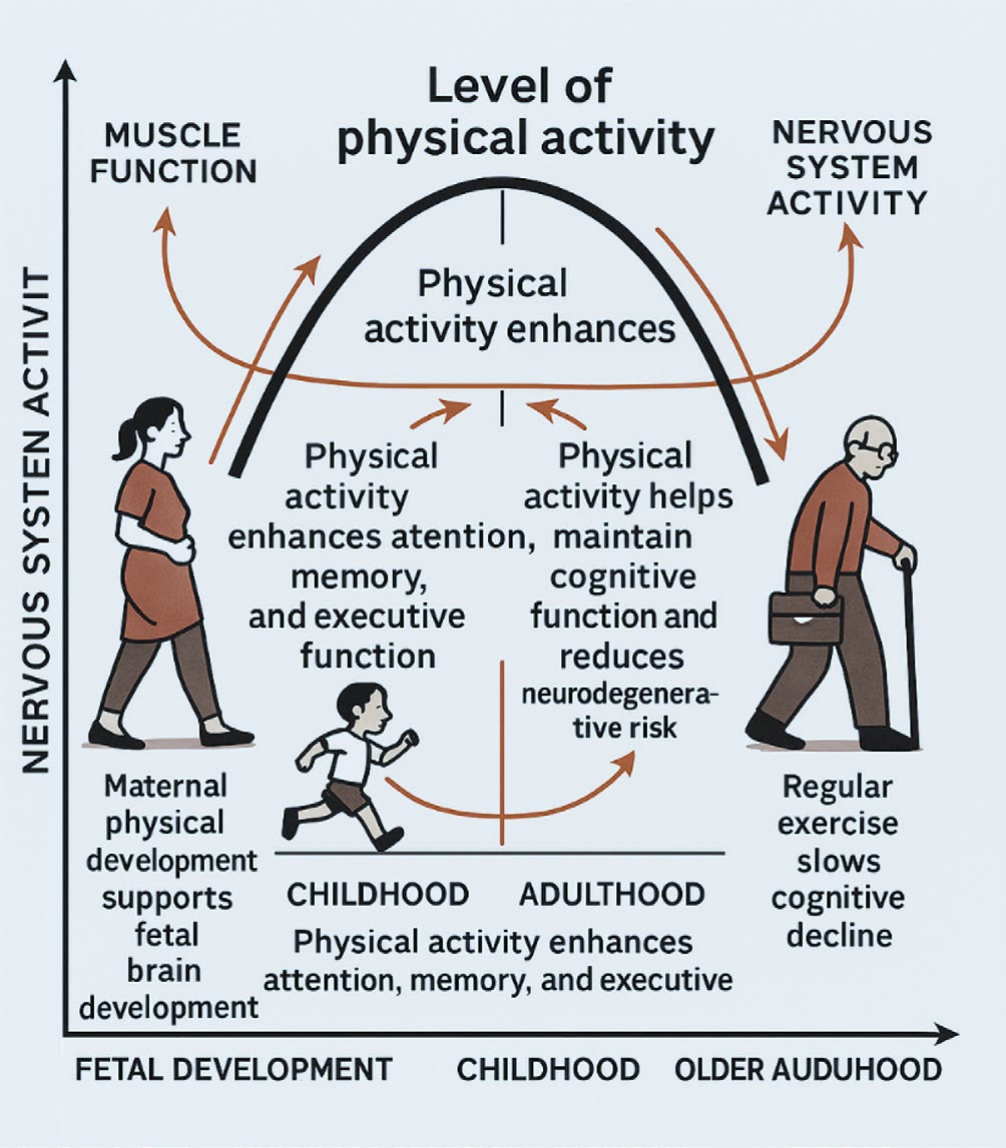
The connection between physical activity and nervous system function across different life stages. During fetal development, maternal physical activity supports healthy brain growth. In childhood, physical activity enhances attention, memory, and EF. During adulthood, physical activity contributes to the preservation of cognitive function and reduces the risk of neurodegenerative disorders. In older adulthood, regular exercise slows cognitive decline and supports muscle and nervous system health.

In older adults, physical activity continues to be a key factor in preserving cognitive function (Erickson et al. 2022). According to the inverted U model and reflected in Figure 1, cognitive function reaches its peak in early adulthood and then gradually declines; however, regular physical activity can slow this decline and help maintain cognitive abilities later in life (Domingos et al. 2021). The benefits of physical activity in this age group are multifaceted, including better physical health, improved mood, and enhanced cognitive function. Erb et al. (2022, 2023) have noted that older adults who engage in regular physical activity are less likely to develop dementia, highlighting its potential as a non‐pharmacological intervention to support cognitive health and mitigate age‐related decline.

The “inverted U” relationship between movement and cognition suggests that there is an optimal level of physical activity that maximizes cognitive function. A balanced approach to physical activity is necessary since too little and too much physical activity can result in less‐than‐ideal cognitive outcomes. This balance is crucial at all stages of life, from fetal development to old age, to promote overall well‐being and cognitive health.

In summary, the interplay between cognitive function and physical activity is complex and dynamic, with significant implications for health and development across the lifespan. By understanding and optimizing this relationship, individuals can enhance their cognitive abilities and overall quality of life.

## 2 The Inverted U‐Shaped Model

### 2.1 Historical Context of Inverted U‐Shaped Trajectories

The historical context of inverted U‐shaped trajectories in developmental psychology is rooted in the observation of cognitive and physical changes across the human lifespan. This concept has been explored extensively to understand how cognitive abilities and physical activities interact and influence each other from early development to old age.

The co‐constructionist model, as outlined by Schaie (2010), proposes that cognitive advancements across adulthood are largely influenced by the accumulation of cultural resources and knowledge over time. This model highlights secular trends in cognitive performance, reflecting differences between cohorts and generations, rather than focusing on within‐individual developmental changes driven by cultural factors. By emphasizing these generational shifts, the model complements lifespan developmental theories by providing a broader context for understanding how cognitive abilities evolve not only through biological aging but also through changing cultural environments.

According to research by Wang et al. (2019), cognitive phenotypes in old age, including dementia, mild cognitive impairment (MCI), and general cognitive decline, are influenced by both intellectual characteristics and genetic predisposition. These findings emphasize that while cognitive decline is a natural part of aging, representing the downward slope of the inverted U, individual trajectories vary based on biological and environmental factors. Physical activity may interact with these factors, potentially buffering the effects of decline and shaping the pace and extent of cognitive aging.

Research shows that cognitive abilities and their underlying neural systems follow diverse and dynamic trajectories throughout life. Petrican et al. (2017), for example, identified age‐related changes in the relationship between brain networks supporting vocabulary and cognitive control. Building on this, Ferguson et al. (2021) found that different components of EF develop along unique paths, some, like cognitive flexibility, peak early and decline later, while others, such as mixing costs, tend to increase with age. Similarly, Salthouse and Davis (2006) highlighted how aging affects the structure and interaction of core cognitive abilities, including fluid intelligence and processing speed. Collectively, these findings highlight the complexity of cognitive aging and underscore the importance of age‐specific interventions.

Li et al. (2024) found that, unlike the inverted U‐shaped trajectory observed in frontoparietal control network regions, the dorsal attention network (DAN), which supports attentional orientation, did not show significant age‐related changes. This finding highlights the differential effects of aging on cognitive networks and underscores the importance of considering these nuances within the inverted U‐shaped framework. Schaie (2010) complements this perspective by emphasizing that while neurobiological factors increasingly drive cognitive aging, secular trends, representing generational shifts in cognitive performance influenced by cultural and environmental factors, also shape cognitive trajectories. Thus, rather than contradicting the inverted U‐shaped model, Schaie's co‐constructionist approach adds a layer of complexity by acknowledging that cohort effects modulate the baseline and magnitude of cognitive function across generations, while the developmental curve itself remains governed by underlying neurobiological processes.

### 2.2 Theoretical Basis and Core Mechanisms

Inverted U‐shaped trajectories in cognitive and physical development are shaped by separate but interacting mechanisms. Biologically, these include synaptic overproduction and pruning, myelination, and neuroplasticity. Psychologically, factors such as motivation, attention, and learning strategies contribute to peak performance during early to middle adulthood. Environmentally, variables such as education, socioeconomic status (SES), and lifestyle habits modulate both the rise and decline in performance. Together, these mechanisms provide a theoretical basis for understanding the rise, optimization, and decline in performance across the lifespan.

Early in life, rapid neurobiological changes, including synaptic proliferation, pruning, and increased myelination, lay the foundation for complex cognitive and motor abilities (Li et al. 2024). These developments reflect evolutionary pressure to optimize function during critical periods of growth (Schaie 2010). Accordingly, adolescence and early adulthood mark a period of peak cognitive engagement. Structuralist lifespan theory describes this stage as a peak of advanced intellectual development (Staudinger 2001), in which highly active control networks support advanced EFs such as planning and decision‐making (Li et al. 2024).

Following this peak, typically occurring between the ages of 25 and 35, depending on the specific cognitive or physical domain (e.g., processing speed, EF, or muscular strength), cognitive and physical abilities gradually decline, becoming increasingly evident from middle age onwards, around the age of 40 to 50. This decline is due to neurobiological changes, including decreased synaptic plasticity, decreased myelination, and degenerative processes, as well as cumulative environmental stressors and lifestyle influences (Ferguson et al. 2021; Morse et al. 2011). The complexity of these changes is highlighted by the need for meta‐analyses to elucidate the lifespan trajectories of cognitive control networks (Li et al. 2024). The decline in cognitive abilities, particularly EF, is not uniform across all components. Some components may show earlier signs of decline, while others remain relatively stable until later in life. Studies with middle‐aged adults are essential to gain a comprehensive understanding of the development and decline of EF throughout adulthood (Ferguson et al. 2021). The onset of cognitive decline during middle age underscores the importance of early intervention strategies targeting modifiable intellectual and psychosocial factors to delay the onset of cognitive disorders and promote healthy brain aging (Wang et al. 2019).

Inverted U‐shaped trajectories in development describe a pattern where certain cognitive and physical abilities improve to a peak point during the lifespan and then decline. This concept is particularly relevant when examining the interplay between movement and cognition from fetal development through old age. Cognitive behavior is influenced by both biological and cultural factors, which interact in a complex fashion throughout the lifespan. The acquisition of pragmatic knowledge is crucial for the development of cognitive mechanics in early life, which is facilitated by physical activity and engagement with the environment (Staudinger 2001). This interaction suggests that the trajectory of cognitive development is not linear but rather follows an inverted U‐shape, where cognitive abilities increase to a peak before declining in later years.

Studies on brain activity linked to cognitive control provide more evidence for the link between physical activity and cognitive function. One study, for example, found a positive correlation between age groups and bilateral prefrontal cortex (PFC) activation; however, the trustworthiness of these results is limited by the small sample size (Li et al. 2024). According to Li et al. (2024), this implies that cognitive control might have an inverted U‐shaped trajectory, peaking in young to middle‐aged people and then dropping after that. Furthermore, a lower incidence of cognitive impairment and dementia in later life is linked to the cumulative effects of multiple intellectual and psychosocial factors across the lifespan, including educational attainment, job complexity, and leisure activities. The concept of an inverted U‐shaped trajectory in cognitive development is further supported by the frequent correlations and potential cumulative impacts of these factors.

This trajectory also aligns with the distinction between fluid and crystallized intelligence. Fluid intelligence, the ability to reason logically and solve novel problems, peaks in early adulthood and declines with age. In contrast, crystallized intelligence, which encompasses knowledge, experience, and skills, tends to increase throughout life and remains stable or declines slightly in older age (Schaie 2010). This dual model highlights the importance of different cognitive abilities and their distinct lifespan trajectories.

### 2.3 Neurotransmitter Modulation

Neurotransmitter modulation plays a crucial role in shaping the inverted U‐shaped trajectories of both cognitive function and physical activity across the lifespan. Neurotransmitters regulate a broad range of cognitive processes and motor behaviors, and the balance among them is essential for sustaining optimal functioning (Teleanu et al. 2022; Alzeer and Alzeer 2023).

Among these, dopamine is especially central to cognitive control. Key functions such as decision‐making and task regulation are mediated by the dorsal striatum, a brain region densely populated with dopamine receptors (de Kloet et al. 2021; Reimer et al. 2024). Notably, dopamine in this region appears to regulate task execution rather than the initiation of cognitive effort. As individuals age, the dopaminergic modulation of the dorsal striatum becomes less efficient (Choi et al. 2023), potentially contributing to a decline in cognitive control observed in later life, consistent with the downward slope of the inverted U.

Additionally, the PFC, involved in higher‐order cognitive functions, exhibits age‐related changes in activation patterns (Ranchod et al. 2023). Activation of the bilateral PFC varies across the lifespan and is closely linked to cognitive control processes. This region can compensate for declines in other areas, such as the hippocampus and sensory cortices, through upregulation, highlighting the dynamic role of neurotransmitter modulation in maintaining cognitive function (Snytte et al. 2024).

Physical activity also interacts with neurotransmitter systems. In older adults with vascular cognitive impairment, aerobic exercise has been shown to influence neuronal activity and improve EF (Li et al. 2024). This suggests that physical activity can modulate neurotransmitter pathways, enhancing cognitive performance. The interplay between physical activity and neurotransmitter modulation is key to understanding the inverted U‐shaped relationship between movement and cognition (Ruiz‐Tejada et al. 2022).

The concept of functional connectivity (FC), which reflects coordinated activity between brain regions, is closely linked to neurotransmitter modulation (Hansen et al. 2022; Keller et al. 2023). Lifespan studies of FC reveal non‐pathological developmental patterns spanning from fetal stages to older adulthood (Edde et al. 2020), underscoring the essential role of neurotransmitter systems in sustaining FC and cognitive health over time. Complementing this, the differentiation‐reintegration hypothesis posits that covariation among cognitive skills decreases from childhood through middle adulthood, then increases in old age (Staudinger 2001). This trajectory aligns with the inverted U‐shaped model and highlights neurotransmitter modulation as a key factor in the evolving interactions among cognitive abilities throughout life.

In summary, neurotransmitter modulation is a fundamental mechanism underlying the inverted U‐shaped trajectories of cognitive function and physical activity. The balance and interaction of neurotransmitters, such as dopamine, in brain regions like the dorsal striatum and PFC are essential for maintaining cognitive control and EF. Moreover, physical activity influences these neurotransmitter systems, thereby enhancing cognitive health. Insights from changes in FC and the differentiation‐reintegration hypothesis further illuminate the complex relationship between neurotransmitter modulation and cognitive function across the human lifespan (Edde et al. 2020; Li et al. 2024; Staudinger 2001).

### 2.4 Hormonal Influences

Hormonal influences play a significant role in shaping the inverted U‐shaped trajectories of movement and cognition throughout the human lifespan. Hormones, as biochemical messengers, regulate various physiological processes, including those related to cognitive function and physical activity. The interaction between hormonal changes and cognitive abilities is particularly evident during critical periods such as puberty, pregnancy, and menopause.

During puberty, the surge in sex hormones such as estrogen and testosterone is associated with significant changes in brain structure and function (Vijayakumar et al. 2021; Liao et al. 2021; Rehbein et al. 2021). These hormonal changes contribute to the development of secondary sexual characteristics as well as influence cognitive processes like spatial abilities and memory (Joue et al. 2024; Killanin et al. 2024). For instance, increased levels of estrogen have been linked to enhanced verbal memory and cognitive flexibility, while testosterone is associated with improvements in spatial abilities (Pauls et al. 2013; Staudinger 2001; Joue et al. 2024). The interplay between these hormones and cognitive functions underscores the importance of hormonal balance in maintaining cognitive health during adolescence.

Pregnancy is another critical period where hormonal fluctuations significantly impact cognitive function. Elevated levels of hormones such as progesterone and estrogen during pregnancy can lead to changes in brain structure, particularly in regions associated with memory and emotional regulation. Some studies suggest that these hormonal changes may contribute to the phenomenon known as “pregnancy brain,” characterized by temporary cognitive impairments such as forgetfulness and reduced attention (Schaie 2010; Staudinger 2001). However, these changes are typically transient, and cognitive function often returns to pre‐pregnancy levels postpartum.

Menopause marks a significant decline in estrogen levels, which can have profound effects on cognitive function and physical activity. The reduction in estrogen is related to an increased possibility of decline in cognitive function and neurodegenerative disorders such as Alzheimer's disease (Jett et al. 2022; Oveisgharan et al. 2023). Estrogen is known to have neuroprotective effects, and its decline during menopause can lead to impairments in attention, memory, and EF (Craik and Bialystok 2006; Li et al. 2024).

In addition to these critical periods, as individuals age, hormonal effects on movement and cognition become more apparent (Sumien et al. 2021; Hegarty et al. 2023). Reduced muscle mass, strength, and cognitive function are linked to the age‐related decline in anabolic hormone levels including growth hormone and insulin‐like growth factor 1 (IGF‐1) (Gökçe et al. 2024). These hormones play crucial roles in maintaining brain plasticity and muscle function, and their decline contributes to the age‐related deterioration in physical and cognitive abilities (Li et al. 2024; Merenstein and Bennett 2022). Interventions aimed at modulating these hormonal levels, such as resistance training and nutritional supplementation, have shown promise in mitigating age‐related declines in both movement and cognition.

### 2.5 Environmental Factors

The “inverted U” relationship is significantly shaped by environmental influences through movement and cognition throughout the lifespan. These factors encompass a wide range of influences, including SES (Dogra et al. 2022), education (Dogra et al. 2022; Sánchez‐Izquierdo and Fernández‐Ballesteros 2021), work complexity (Parker et al. 2021), and mental activity (Alvares Pereira et al. 2022), all of which can significantly impact cognitive function and physical activity levels.

The beginning of cognitive disorders like dementia can be postponed by life‐course intellectual stimulation, such as in lifelong education and work complexity (Majoka and Schimming 2021; Wang et al. 2023). This implies that cognitive health may benefit from lifelong participation in cognitively challenging activities. Additionally, several cardiometabolic risk factors, like high blood pressure, obesity, and high cholesterol, have been found to operate as risk factors for dementia and late‐life cognitive impairment during particular time frames by systematic reviews (Peters et al. 2021; Jones et al. 2024). When these risk factors manifest in middle age and young adulthood as opposed to later in life, they have a particularly significant impact.

Beyond intellectual stimulation, regular physical activity has been shown to play a crucial role in mitigating age‐related cognitive decline. Different types of exercise, including aerobic, resistance, and coordination training, have demonstrated benefits for brain health by improving neuroplasticity, vascular function, and reducing inflammation. This aligns with the paper's focus on optimal physical activity as a key factor in maintaining cognitive function throughout life, complementing the protective effects of lifelong cognitive engagement.

Physical exercise and cognitive performance have a complicated relationship that is impacted by several environmental influences. For example, there is no evidence of a negative correlation between midlife physical activity and the risk of dementia and cognitive decline in later life, according to the Whitehall II study (Marmot et al. 2013; Machado‐Fragua et al. 2023). A two‐year program of moderate‐intensity physical activity did not enhance global or domain‐specific cognitive performance, according to the findings of the Lifestyle Interventions and Independence for Elders Study (Marsh et al. 2013; Wang et al. 2019). These results underline how important it is to have a comprehensive understanding of how physical activity affects cognitive health in conjunction with other environmental factors.

In addition to intellectual and physical activities, other environmental factors such as SES and IQ also play a role in cognitive aging. Studies have shown that SES is not directly related to EF components when controlling for IQ and age. This suggests that while SES may influence cognitive development indirectly, its direct impact on EF is limited. However, IQ appears to hold a defense against aging‐related EF reductions, indicating that cognitive abilities developed early in life can have long‐lasting effects on cognitive health.

The development of EF across the lifespan is influenced by a continuous interplay of environmental factors. By examining a continuous age sample, researchers have highlighted changes in EF that emerge throughout adulthood, rather than focusing solely on the onset of old age. This approach allows for a more comprehensive understanding of how cognitive abilities evolve and the importance of considering all ages when studying cognitive changes (Ferguson et al. 2021). Older adult EF performance depends mainly on left alpha activity and anterior–posterior theta‐power dynamics during working memory retention intervals. Age‐related decreases in alpha activity may result in decreased frontal‐parietal alpha coherence and fronto‐parietal cortical connection, which have been linked to AD neurophysiology (Meiron et al. 2021). A rapid decline in alpha power may signal the final stage in the inverted U‐shaped trajectory for EF and everyday WM functioning related to decline in fronto‐parietal white matter integrity in aging people and neurodegenerative disorders.

Moreover, the brain's functional modularity, which changes throughout development, is also affected by environmental factors. Studies have shown that functional brain networks become less differentiated with age, following an inverted U‐shaped developmental trajectory. This indicates that environmental influences on brain development can have long‐term effects on cognitive function (Gozdas et al. 2019).

## 3 Cognitive and Motor Development Across the Lifespan

Cognitive and motor developments are deeply intertwined processes that evolve together across the human lifespan. Traditionally studied as separate domains, research increasingly reveals that movement and cognition are co‐dependent systems, influencing and shaping each other from the earliest stages of development through aging. The dynamic interaction between motor behavior and cognitive function underpins learning, perception, attention, memory, and EFs, while also being shaped by them in return.

This chapter explores the parallel and interconnected development of motor and cognitive abilities across distinct life stages: from prenatal development and early infancy, through childhood and adolescence, and into adulthood and old age. We highlight key neurodevelopmental milestones, behavioral markers, and neurobiological mechanisms that illustrate how movement and cognition co‐evolve. Special attention is given to critical periods where changes in one domain significantly influence the other, for example, how early motor exploration supports cognitive growth, or how cognitive decline may manifest in motor performance in aging.

By examining this co‐developmental trajectory through the lens of the “inverted U” framework, we offer an integrated perspective that underscores the importance of balance in physical and cognitive engagement throughout life.

Cognitive development across the lifespan is a multifaceted process influenced by lifestyle, environmental, as well as genetic factors. The trajectory of cognitive abilities often follows an “inverted U” pattern, where cognitive performance improves during childhood and early adulthood, peaks in midlife, and then declines in older age (Brown et al. 2021).

Different brain networks exhibit distinct lifespan trajectories (Edde et al. 2021; Snyder et al. 2021). For instance, the frontoparietal control network and the cingulo‐opercular network (CON) show different patterns of age‐related changes (Han et al. 2023; Xia et al. 2022), reflecting their unique roles within the brain's functional hierarchy. The DAN, which underlies attention, maintains a relatively stable function throughout aging, contrasting with the inverted U‐shaped trajectory observed in the frontoparietal control network (Wong et al. 2023; Lee et al. 2023).

EFs, such as inhibitory control (Kang et al. 2022; Treacy et al. 2025), working memory (Kronovsek et al. 2021), cognitive flexibility (Amelchenko et al. 2023), and planning (Ferguson et al. 2021), also exhibit age‐related changes. These functions are crucial for goal‐directed behavior and problem‐solving. Studies have shown that these EFs continue to evolve and differ across the lifespan, with specific tasks revealing variations in performance among different age groups (Ferguson et al. 2021). Research findings on working memory and response inhibition demonstrate variability: some report increased recruitment of brain regions with age, while others note decreases or more complex patterns. This diversity highlights the complexity of cognitive development and decline and underscores the need for a multidimensional approach to understanding the inverted U‐shaped trajectories of EFs.

Cognitive aging is further influenced by intellectual and genetic factors. Long‐term cognitive training exercises have been shown to improve cognitive function or delay cognitive decline among older healthy adults (Butler et al. 2018), suggesting that cognitive benefits observed in longitudinal studies may partly result from reverse causality.

The concept of cognitive reserve, the brain's resilience to neuropathological damage, plays a significant role in mitigating age‐related cognitive decline. Cognitive reserve refers to the brain's ability to efficiently utilize existing neural networks or recruit alternative networks to cope with damage. Engaging in leisure activities that combine physical, social, and mental components has been shown to reduce the risk of dementia more effectively than participating in any single type of activity. This cumulative effect highlights the importance of a multifaceted approach to maintaining cognitive health in later life. Moreover, physical activity at any stage of life is associated with a decreased risk of dementia and late‐life cognitive impairment, especially when it occurs in early life and middle age (Wang et al. 2019).

The relationship between cognitive development and physical activity is complex and bidirectional. Cognitive engagement through cultural activities and educational attainment can help maintain crystallized intelligence, while fluid intelligence is more susceptible to decline due to chronic diseases. The dual intelligence model posits that fluid intelligence underlies the development of crystallized intelligence, highlighting the interconnection of different cognitive domains (Schaie 2010).

### 3.1 Prenatal Brain and Motor Development

Motor development across the lifespan is a complex interplay of physical and cognitive processes that evolves from fetal development to old age. This development is characterized by an “inverted U” relationship, where motor skills and cognitive functions peak at certain life stages and decline at others.

Motor skills begin to emerge as early as the fetal stage, where movements are critical for the development of the musculoskeletal and nervous systems, laying the groundwork for later motor function (Leisman et al. 2024). As children grow older, their motor skills become more sophisticated, influenced by both genetic and environmental factors. The development of EF, such as cognitive control, follows a similar trajectory, peaking in young adulthood (Erb et al. 2022, 2023; Leisman et al. 2024). This period is characterized by high levels of brain activity in cognitive control, which is essential for complex motor tasks.

Prenatal motor development is a critical phase in the human lifespan, characterized by the emergence and maturation of motor functions even before birth. During this period, the fetal brain exhibits a modular organization, which includes highly interconnected regions known as networks (Leisman et al. 2024). These networks are essential for the development of motor functions and are present before birth, indicating the early establishment of FC in the brain.

### 3.2 Early Fetal Activity and Neural Networks

**FIGURE 2:**
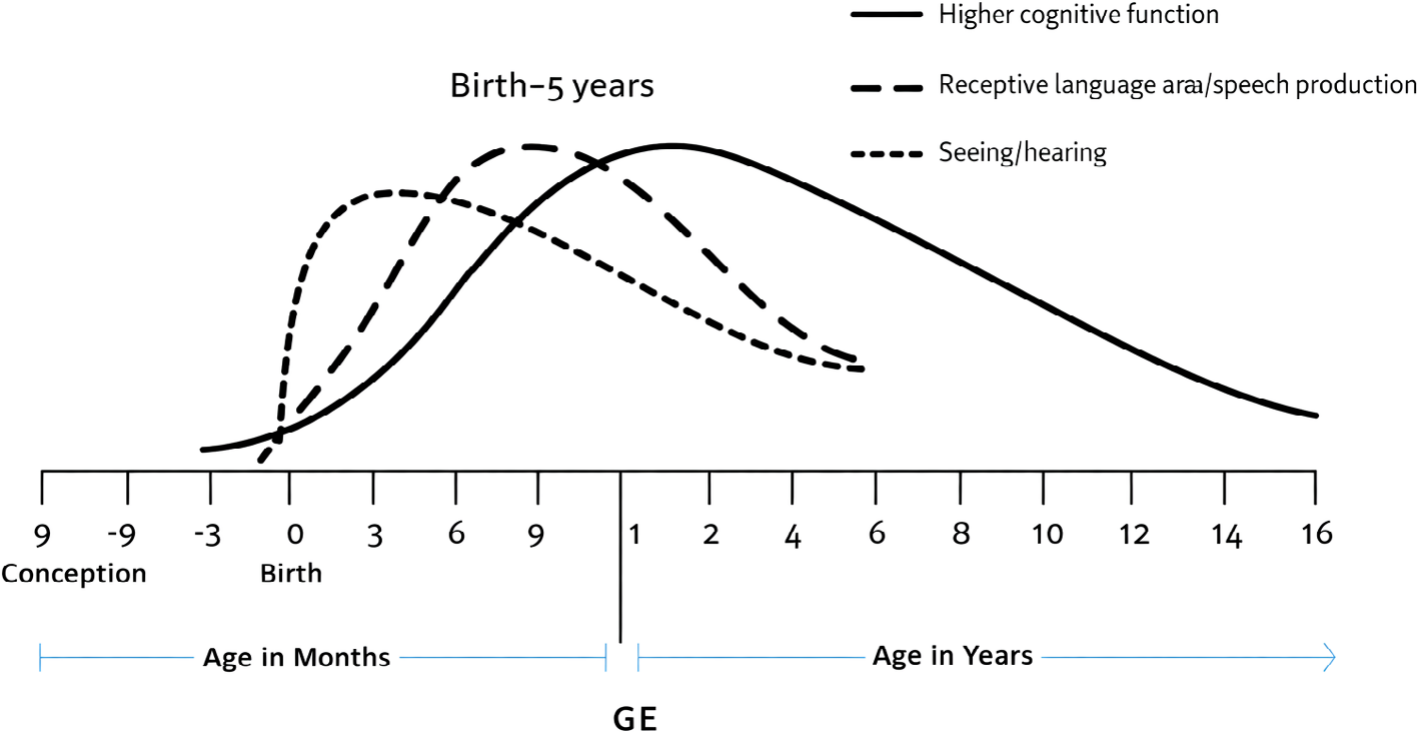
Synaptic density during early brain development, illustrating the overproduction and subsequent pruning of neural connections.

Neural network formation in utero is a dynamic process starting early in fetal development. Neural progenitor cells proliferate extensively to form the neural tube, the precursor of the central nervous system (Belmonte‐Mateos and Pujades 2022; Figure 2). This stage establishes the brain's basic architecture, including primary regions and initial neural circuit patterning (Morse et al. 2011).

As gestation progresses, progenitor cells differentiate into various neurons and glial cells under genetic and environmental influences (Morse et al. 2011; Belmonte‐Mateos and Pujades 2022). This differentiation forms specialized neural circuits crucial for sensory, motor, and cognitive functions (Li et al. 2024; Morse et al. 2011).

**FIGURE 3:**
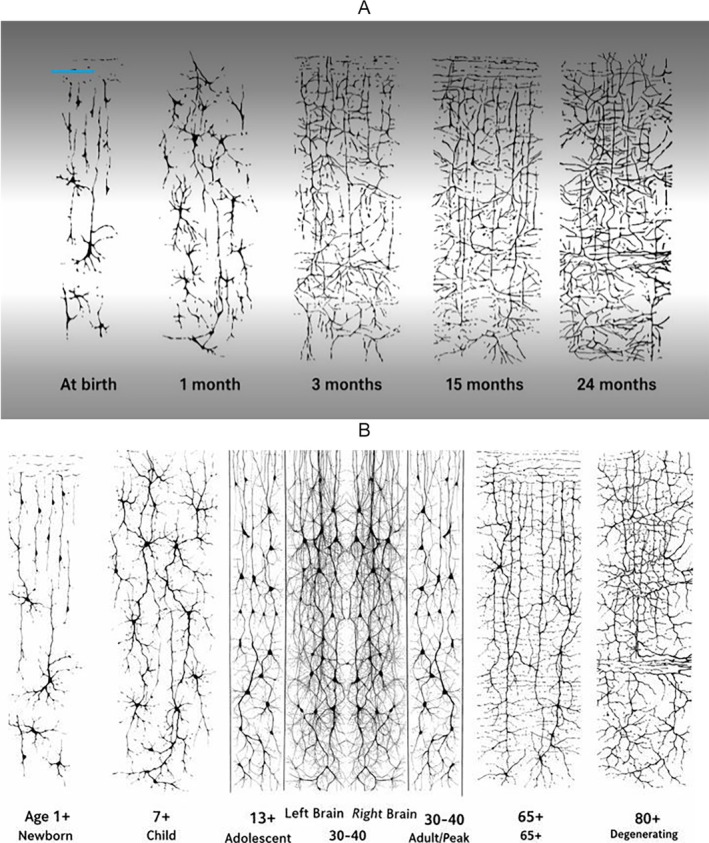
Development of exuberant neural connectivities (A) from birth to 24 months postpartum and (B) across the lifespan.

Of the key aspects of neural network formation in utero is the establishment of synaptic connections between neurons. Synaptogenesis, the formation of synapses represented in Figure 3, begins in the second trimester and continues throughout fetal development (Sarnat 2023). This process is highly dynamic, with synapses being formed, strengthened, or pruned based on neural activity and environmental inputs. The activity‐dependent nature of synaptogenesis highlights the importance of early sensory and motor experiences in shaping the developing brain.

The development of neural networks in utero is also influenced by the formation of FC between different brain regions. FC refers to the coordinated activity of different brain regions that work together to support various cognitive and motor functions. Studies have shown that even in utero, FC patterns resemble those seen in the mature brain (Edde et al. 2021; Eyre et al. 2021; Leisman et al. 2024). These early patterns of connectivity are thought to be crucial for the development of higher‐order cognitive functions and the integration of sensory and motor information.

Neural network formation in utero involves dynamic phases of rapid synapse formation followed by selective pruning, which refines neural circuits to enhance efficiency and functionality. While this process shows an initial surge in connectivity, pruning is a critical developmental step, not a suboptimal decline. After this peak, there may be gradual decreases in regenerative capacity or plasticity later in life, but pruning itself supports maturation and optimal brain function (DuPre and Spreng 2017; Edde et al. 2020; Li et al. 2024).

Edde et al. (2020) indicated that the inverted U‐shaped trajectory of neural development is also evident in the age‐related changes in brain connectivity. Early in development, there is an increase in connectivity within and between different brain networks, followed by a decrease in connectivity as the brain matures and becomes more specialized. This pattern is thought to reflect the brain's transition from a highly plastic and adaptable state to a more stable and efficient configuration.

Studies utilizing independent component analysis (ICA) have documented the spatiotemporal elements of brain activity during pregnancy, demonstrating substantial cross‐hemisphere and intralobular connectivity in normally growing fetuses. Specifically, bilateral occipital functional components, including the primary and secondary visual cortices, have been noted. Additionally, bilateral components in the medial and lateral prefrontal regions, as well as unilateral components in the temporal lobe, predominantly on the right side, have been reported (Jakab 2019; Edde et al. 2021). These findings suggest that the prenatal brain is already organizing itself in a way that supports future motor and cognitive functions.

The development of motor functions in the prenatal stage is not isolated but is closely linked with the overall growth of the brain's structure. Both gray and white matter volumes have an inverted U‐shaped trajectory with age, according to recent large‐cohort studies (Li, Petersen, et al. 2023). Gray matter volume peaks in early adolescence, while white matter volume peaks in young adulthood (Brouwer et al. 2022; Bethlehem et al. 2022). This trajectory indicates that the foundational structures necessary for motor development are established early and continue to evolve throughout childhood and adolescence.

Furthermore, the modular organization of the fetal brain, as revealed by graph‐theory studies, underscores the importance of early neural connectivity in supporting motor development. The presence of hub regions, which are highly connected nodes within the brain network, before birth, highlights the brain's preparedness for complex motor functions postnatally (Edde et al. 2020; Leisman et al. 2024). These hub regions are crucial for integrating sensory and motor information, facilitating coordinated movements, and laying the groundwork for future cognitive and motor skills.

**FIGURE 4:**
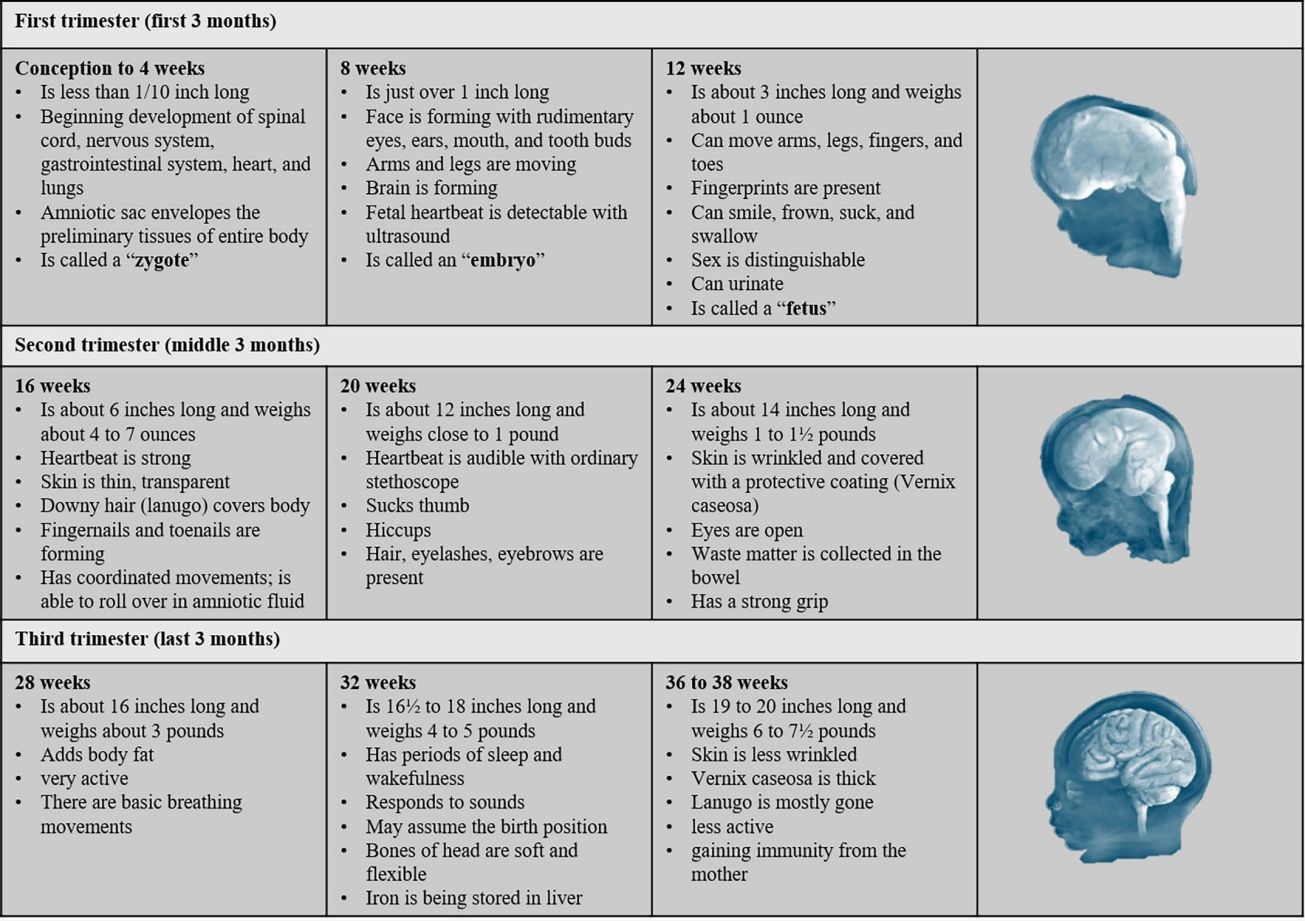
Three trimesters of prenatal development.

Fetal brain development and movement patterns are intricately linked, with significant implications for cognitive and motor functions throughout the human lifespan. During the fetal period, the brain undergoes rapid growth and differentiation, establishing the foundational neural circuits that will support future cognitive and motor abilities (as exemplified in Figure 4). Brain hubs, which are critical for efficient neural communication, begin to emerge during this period, primarily located in primary sensorimotor, auditory, and motor regions. These early developments are crucial as they set the stage for subsequent brain maturation and functional specialization.

The establishment of these neural hubs is influenced by both genetic and environmental factors, including the fetus's movements. Spontaneous movements in utero, such as kicking and stretching, are not merely reflexive actions but play a role in shaping the developing brain. These movements stimulate the sensorimotor cortex, promoting the growth of neural connections and the refinement of motor pathways (Edde et al. 2020). This activity‐dependent development underscores the importance of early motor experiences in the formation of a functional and adaptable nervous system.

As the fetus grows, the complexity of its movements increases, reflecting the maturation of the central nervous system. This progression is marked by a transition from simple, repetitive movements to more coordinated and purposeful actions. The development of the DAN and other neural systems during this period supports this increasing complexity (Onofrj et al. 2022). The DAN, for instance, is involved in the regulation of attention and motor planning, and its maturation is essential for the coordination of more sophisticated motor behaviors (Karolis et al. 2023).

The relationship between fetal movement patterns and brain development is further evidenced by studies on structural covariance. These studies show that regions involved in motor control, such as the motor and visual cortices, exhibit increased structural connectivity during early development. This connectivity decreases linearly with age, suggesting that the foundational neural architecture established during the fetal period undergoes continuous refinement and reorganization throughout life (DuPre and Spreng 2017).

Moreover, different brain regions exhibit distinct developmental trajectories, with higher‐order regions, including the PFC, maturing later and declining earlier than primary sensorimotor areas (Li et al. 2024). This differential development highlights the importance of early motor experiences in shaping the brain's functional architecture. The PFC, which is crucial for cognitive control and EF, benefits from the early establishment of robust sensorimotor networks, facilitating the integration of motor and cognitive processes.

Functional MRI (fMRI) studies provide additional insights into the relationship between fetal brain development and movement patterns. These studies reveal that age‐related differences in brain activity can be inferred from the blood‐oxygen‐level‐dependent (BOLD) signal, reflecting neural activity during both task performance and rest (Merenstein and Bennett 2022). The BOLD signal patterns observed in fetuses and young children indicate that early motor experiences contribute to the development of efficient neural networks, which support cognitive and motor functions later in life.

The concept of an inverted U‐shaped trajectory in cognitive and motor development is supported by findings that EF, including inhibition, working memory and cognitive flexibility, follows a pattern of improvement during childhood and decline in later adulthood (Ferguson et al. 2021). This trajectory underscores the importance of early brain development and movement patterns in establishing the neural foundations for lifelong cognitive and motor abilities.

Fetal brain development and movement patterns are closely intertwined, with early motor experiences playing a crucial role in shaping the neural architecture that supports cognitive and motor functions throughout life. The emergence of brain hubs, the maturation of neural networks, and the establishment of structural connectivity during the fetal period are all influenced by the fetus's movements, highlighting the importance of a dynamic interplay between motor activity and brain development. Prenatal motor development is a complex process that involves the early establishment of functional brain networks and the maturation of brain structures essential for motor and cognitive functions. The modular organization of the fetal brain, the presence of hub regions, and the inverted U‐shaped trajectory of brain volume growth all contribute to the foundation of motor development (Turk et al. 2019; De Asis‐Cruz et al. 2021; Kostović et al. 2021). Understanding these early developmental processes is crucial for comprehending how motor and cognitive functions evolve throughout the human lifespan.

In summary, the formation of neural networks in utero is a highly dynamic and complex process that involves the proliferation and differentiation of neural cells, the establishment of synaptic connections, and the development of FC. This process follows an inverted U‐shaped trajectory, with periods of rapid growth and connectivity followed by phases of pruning and refinement. These early stages of neural development are crucial for laying the foundation for cognitive and motor functions that will continue to develop and mature throughout life (Edde et al. 2020; Morse et al. 2011).

### 3.3 Early Fetal Motor Activity and Primitive Reflexes

Early motor activity and reflexes before birth are critical components of fetal brain development and movement patterns. These early movements are not only indicative of the developing neuromuscular system but also are involved in forming the brain's functional and structural maturation.

Primitive reflexes are innate, automatic motor responses that emerge during fetal development and are typically present at birth. Examples include the Moro reflex, rooting reflex, asymmetric tonic neck reflex (ATNR), and grasp reflex (Sigafoos et al. 2021; Melillo et al. 2022; Leisman et al. 2023). These reflexes, mediated by the brainstem and spinal cord, support vital functions such as feeding, protection, and initial motor exploration. They play a critical role in early sensorimotor integration, laying the foundation for more complex voluntary motor behaviors observed later in infancy (Needlman and Silverstein 2004).

The importance of early motor activity extends beyond reflex integration. Repetitive and rhythmic fetal movements contribute to the organization of neural circuits involved in motor control, promote synaptogenesis, and support the FC of the brain. Sensory feedback from movement is believed to stimulate glial proliferation and gene expression related to neuronal growth (Kuiper et al. 2019; Fields and Stevens‐Graham 2002), ultimately enhancing the development of higher‐order motor regions. These foundational processes have long‐term implications, as both the quality and quantity of fetal movements are predictive of motor and cognitive outcomes in early childhood (Edde et al. 2020).

Primitive reflexes do not conform to the classic inverted U‐shaped developmental trajectory. Rather, they are highly active in early development, suppressed with cortical maturation, and may reappear later in life under pathological conditions. This positions them as functional markers at both ends of the neural development curve, during early immaturity and later‐stage cognitive decline (Edde et al. 2020; Leisman et al. 2023).

## 4 Motor and Cognitive Trajectories Across the Lifespan

### 4.1 Motor Development in Early Childhood

Childhood motor milestones represent critical stages in the development of motor skills, necessary for a child's overall cognitive development and physical growth. These milestones include a series of progressive achievements such as sitting, crawling, standing, and walking, which typically occur in a predictable sequence during early childhood. The development of these motor skills is influenced by a combination of genetic, environmental, and sociocultural factors.

The co‐constructionist model suggests that both neurobiological and sociocultural factors play a significant role in the differential impact on cognitive abilities and motor development. This model highlights the importance of considering both intrinsic and extrinsic influences when examining childhood motor milestones (Schaie 2008, 2010). For instance, it has been demonstrated that midlife work complexity and early life educational attainment impact late‐life cognitive phenomena, indicating that early motor development can have long‐term implications for cognitive health (Wang et al. 2019).

As children grow, their motor skills become more refined and complex. This progression is closely linked to the maturation of the brain and the development of cognitive control. Cognitive control, which includes processes such as attention, inhibition, and working memory, increases in speed, power, and complexity from infancy to adolescence and young adulthood (Craik and Bialystok 2006). This development is necessary for the successful acquisition of motor skills, as it allows children to plan, execute, and adjust their movements effectively.

Toddlers' growth in gross motor skills, which include the development of abilities like walking, running, jumping, and climbing, is a crucial component of early life milestones. Both biological maturation and environmental variables influence these abilities, which serve as the basis for increasingly intricate motions. During the toddler years, children typically exhibit significant advancements in their gross motor abilities. This period is marked by rapid growth and the refinement of motor coordination. For instance, toddlers begin to walk independently, which is a major milestone that usually occurs around 12–18 months of age. This newfound mobility allows them to explore their environment more freely, which in turn promotes further motor development.

The development of gross motor skills is not uniform across all children, as individual differences can be attributed to genetic, environmental, and experiential factors. For example, some toddlers may develop these skills earlier or later than their peers, depending on their unique developmental trajectory. The inverted U‐shaped development pattern observed in certain cognitive and motor abilities suggests that there may be periods of regression followed by significant improvements, reflecting the dynamic nature of early childhood development (Pauls et al. 2013). Studies have indicated that the maturation of neural pathways and the increasing myelination of motor neurons play a crucial role in the enhancement of gross motor skills during this stage. The brain's plasticity allows for the adaptation and strengthening of motor circuits in response to physical activity and practice. This neural development is essential for the coordination and control required for complex movements (Petrican et al. 2017).

Moreover, the interaction between cognitive and motor development is evident in the way toddlers learn and refine their gross motor skills. Cognitive processes such as attention, problem‐solving, and memory is integral to mastering new motor tasks. For instance, a toddler learning to climb stairs must not only have the physical strength and balance but also the cognitive ability to plan and execute the sequence of movements required. The role of environmental factors, including parental support and opportunities for physical activity, cannot be overstated. Toddlers who are provided with a stimulating environment that encourages movement and exploration are likely to develop their gross motor skills more effectively. Activities such as playing on playground equipment, participating in structured physical activities, and engaging in free play are all beneficial for motor development (Zimmermann and Meier 2006). In addition to environmental influences, the child's motivation and self‐efficacy also play a significant role in the development of gross motor skills. Toddlers who are confident in their abilities and motivated to explore their surroundings are more likely to practice and refine their motor skills. This intrinsic motivation is often supported by positive reinforcement from caregivers and peers (Sheffler et al. 2022).

Additionally, language plays an important role in the development of motor abilities. As children grow, language becomes a key contextual factor influencing both cognitive and motor development. Studies have shown that more mature individuals exhibit greater connectivity between the ventral attention network (VAN) and the CON, which supports sustained control and alertness during tasks (Petrican et al. 2017). These findings suggest a dynamic interaction between language development and motor skills, where advancements in one domain may support progress in the other, without necessarily following a specific inverted U‐shaped trajectory.

In infancy, motor development milestones such as reaching, grasping, and crawling are not only indicators of physical growth but also play a crucial role in cognitive development. These motor skills enable infants to explore their environment, which in turn stimulates cognitive processes such as perception, attention, and memory. For instance, the act of reaching for and manipulating objects helps infants develop an understanding of object permanence and spatial relationships (Craik and Bialystok 2006; Ferguson et al. 2021). It is the development of sensorimotor integration that in part supports cognitive development in infancy and early childhood.

Sensorimotor integration in infants is a critical aspect of early development, encompassing the coordination of sensory inputs and motor outputs. This process is fundamental for the acquisition of motor skills and cognitive functions. During infancy, the brain undergoes rapid growth and development, which is reflected in the increasing complexity of sensorimotor integration. Research indicates that the development of sensorimotor integration follows a trajectory that can be described by an inverted U‐shaped pattern. This pattern suggests that sensorimotor abilities improve significantly during early childhood, peak at a certain point, and then gradually decline with age (Li et al. 2024). This trajectory is influenced by both biological maturation and environmental interactions.

In the early stages of life, infants rely heavily on sensory inputs to guide their motor actions. For instance, visual and auditory stimuli play a crucial role in the development of hand‐eye coordination and other motor skills. The integration of these sensory inputs with motor responses is essential for tasks such as reaching, grasping, and manipulating objects. Studies have shown that infants who are exposed to a rich sensory environment tend to develop better motor skills compared to those with limited sensory experiences (Gozdas et al. 2019; Petrican et al. 2017).

The brain regions involved in sensorimotor integration, such as the parietal and frontal cortices, show significant activity during infancy. These regions are responsible for processing sensory information and coordinating motor responses. FC within these brain networks is crucial for efficient sensorimotor integration. For example, the development of visual and auditory networks (AUDs) is closely linked to the ability to perform complex motor tasks (Edde et al. 2020; Petrican et al. 2017).

As infants grow, their sensorimotor integration becomes more refined, allowing for more precise and coordinated movements. This refinement is facilitated by the strengthening of neural connections and the pruning of unnecessary synapses, a process that enhances the efficiency of neural networks. The dynamic nature of these changes underscores the importance of early experiences in shaping sensorimotor development (Craik and Bialystok 2006; Staudinger 2001). Furthermore, the interaction between cognitive and motor functions is evident in the development of EF, such as cognitive flexibility, updating, and inhibition. These EFs are crucial for goal‐directed behavior and are supported by the integration of sensory and motor information. The development of these functions follows a similar inverted U‐shaped trajectory, highlighting the interconnectedness of cognitive and motor development (Ferguson et al. 2021; Li et al. 2024).

Creeping and crawling are fundamental stages in the development of gross motor skills in toddlers. These stages are crucial as they lay the foundation for more complex movements and cognitive functions later in life. During the creeping stage, infants typically move on their bellies, using their arms and legs to propel themselves forward. This stage is often followed by crawling, where the infant moves on hands and knees, which requires more coordination and strength.

The maturation of the brain's functional networks is intimately related to the development of these motor skills. By approximately 6 months of age, hubs have progressively spread from their initial location in basic sensory networks to areas including the cingulate cortex, temporal lobe, and thalamus. Higher‐order networks, such as the lateral PFC, insula, and parietal areas, show new hubs by 9 months. The coordination needed for creeping and crawling depends on the gradual integration of functional links between remote areas and network connectivity.

As infants begin to crawl, they gain recognition abilities that rely on local information. Simultaneously, associations are formed between different classifications, providing more information through priming. This process helps refine and reorganize pattern recognition abilities, although it may temporarily cause interference in multi‐modal tasks (Morse et al. 2011). The ability to crawl not only enhances physical coordination but also supports cognitive development by enabling infants to explore their environment more effectively. The emergence of high‐order networks during this period is also associated with significant developmental milestones. For instance, the DMN, which is linked to self‐reflection and social cognition, becomes more prominent. The PCC, central to emotional processing, also plays a role during this stage. These developments coincide with the time when children become independently mobile and start to exhibit language skills.

Furthermore, despite variations in FC strength, the architecture of large‐scale functional networks in childhood seems to remain consistent until young adulthood. This stability implies that early adolescence network refinement has an impact on the strength of connectivity both within and between networks (Edde et al. 2020). The refinement of these networks is crucial for the coordination and balance required in crawling and other gross motor skills.

Curiosity also plays a significant role in the development of motor skills. From infancy, curiosity drives exploration and attention allocation to stimuli in the environment. This intrinsic motivation encourages infants to engage in activities like crawling, which in turn supports their cognitive and physical development (Sheffler et al. 2022). The interaction between curiosity and motor skill development highlights the importance of a balanced approach to promoting overall well‐being.

Children's motor skills become increasingly sophisticated as they grow from infancy to early childhood. Children have greater possibilities to engage in more complex forms of play and interact with their environment while they are walking, running, and using other forms of mobility. Improvements in EF as well as other cognitive domains, including working memory, cognitive flexibility, and inhibitory control, are linked to this increased physical exercise. According to Ferguson et al. (2021), inhibitory control can be divided into effortful and automatic inhibition, both of which are essential for goal‐directed behavior and problem‐solving.

In early life, walking and balance control are essential for toddlers' development of gross motor abilities. Children's motor skills, which are crucial to their entire physical and cognitive development, alter significantly throughout this period. The emergence of walking typically occurs around the first year of life, marking a major milestone in a child's motor development. This process involves the coordination of various muscle groups and the integration of sensory information to maintain balance and navigate the environment.

The development of walking and balance control is influenced by the maturation of the brain's motor and sensory networks. According to Edde et al. (2020), the primary sensory and higher‐order networks in the brain present connections with subcortical regions, particularly the thalamus, during the first year of life. These connections become more distributed and strengthen with age, supporting the development of motor skills such as walking. The thalamic‐primary sensory network, in particular, plays a significant role in the integration of sensory information necessary for balance control. As children begin to walk, they must learn to coordinate their movements and maintain stability. This requires the development of both static and dynamic balance control. Static balance involves maintaining a stable position while standing still, whereas dynamic balance involves maintaining stability while moving. The ability to control balance dynamically is particularly important for walking, as it allows children to adjust their posture and movements in response to changes in their environment.

Research indicates that the development of balance control is a gradual process that continues throughout early childhood. Gozdas et al. (2019) note that brain maturation continues until the early twenties, suggesting that the development of motor skills and balance control continues well beyond the toddler years. However, the foundational skills necessary for walking and balance are established during early childhood, providing a basis for more complex motor abilities later in life.

The development of walking and balance control is also influenced by the child's environment and experiences. Opportunities for physical activity and exploration are essential for the development of these skills. The emergence of the primary‐to‐higher‐order network sequence underlying basic network organization becomes stronger during adolescence and stabilizes in maturity. This sequence highlights the importance of early experiences in shaping the development of motor skills and balance control. In addition to environmental factors, individual differences in motor development can also play a role. Some children may develop walking and balance control earlier or later than their peers, depending on a variety of factors, including genetic predispositions and overall health. Petrican et al. (2017) note that large‐scale aging research can employ the creation of more useful and user‐friendly direct measures of cerebrovascular reactivity, which could also be adapted to study motor development in early childhood. Overall, the development of walking and balance control in early childhood is a complex process that involves the integration of sensory and motor information, the maturation of neural networks, and the influence of environmental and individual factors. Understanding these processes is essential for promoting healthy motor development and identifying potential areas for intervention in children who may be at risk for motor delays.

The development of these EFs is supported by changes in brain structure and function. The frontoparietal control network, which encompasses areas like the inferior frontal gyrus and inferior parietal lobule, has been shown to exhibit an inverted U‐shaped trajectory throughout the lifespan (Li et al. 2024). This trajectory reflects the dynamic nature of brain development, with periods of rapid growth and reorganization during early childhood that are critical for the maturation of cognitive control abilities.

Furthermore, the importance of context in supporting cognitive development cannot be overstated. The environment in which a child grows up, including the availability of opportunities for physical activity and cognitive stimulation, plays a significant role in shaping their developmental trajectory. For example, children who have access to safe play spaces and engaging educational activities are more likely to develop strong motor and cognitive skills (Craik and Bialystok 2006; Li et al. 2024).

The relationship between movement and cognition during early childhood is also influenced by individual differences in brain laterality (Leisman et al. 2022). Studies have shown that the laterality of brain activity, or the degree to which certain cognitive functions are lateralized to one hemisphere of the brain, changes with age and can impact cognitive control and attention (Leisman et al. 2022; Li et al. 2024).

In summary, the milestones in movement and cognition during infancy and early childhood are deeply interconnected. Physical activity not only supports motor development but also enhances cognitive function by providing opportunities for exploration and learning. The brain undergoes significant changes during this period, with the development of EF and the frontoparietal control network following an inverted U‐shaped trajectory. The environment and individual differences in brain laterality further influence this complex interplay, highlighting the need for a holistic approach to supporting children's development. The concept of the “inverted U” relationship between movement and cognition is particularly relevant when examining childhood motor milestones. This relationship posits that both physical activity and cognitive function can influence each other, with an optimal balance promoting overall well‐being. During childhood, engaging in physical activities that challenge motor skills can enhance cognitive development, while cognitive tasks that require motor coordination can improve motor abilities. This bidirectional relationship underscores the importance of providing children with opportunities for both physical and cognitive activities to support their development. Furthermore, the inversion of developmental processes during aging, as described in developmental models of neuroanatomy, suggests that the latest matured regions are the first to deteriorate (Edde et al. 2020). This highlights the importance of early motor development, as the skills acquired during childhood can have lasting effects on cognitive and motor functions throughout the lifespan.

### 4.2 Motor Development in Middle Childhood and Adolescence

Middle childhood and adolescence represent critical periods in the development of cognitive and motor functions. During these stages, the interplay between physical activity and cognitive abilities becomes increasingly evident, reflecting the “inverted U” relationship that characterizes this interaction across the lifespan. In middle childhood, children experience significant improvements in cognitive flexibility and working memory. These cognitive advancements are partly due to the maturation of the frontal lobes, which are among the last cortical areas to mature and are crucial for EF. The development of these brain regions supports the child's growing ability to overcome attentional inertia and enhances cognitive control, allowing for more complex and flexible thinking.

Advancements in fine and gross motor skills during middle childhood and adolescence are critical for overall development and well‐being. These skills are influenced by a combination of genetic, environmental, and cognitive factors, which interact in complex ways throughout these developmental stages. During middle childhood, children experience significant improvements in both gross and fine motor skills. Writing, painting, and handling small objects all require fine motor skills, which are the coordination of small muscles, especially in the hands and fingers. Conversely, gross motor abilities are essential for activities like balancing, sprinting, and jumping, because they necessitate the coordination of larger muscle groups. The development of these skills is not linear but follows a trajectory that can be influenced by various factors, including physical activity and cognitive development.

Studies have indicated that the development of motor skills is closely linked to cognitive functions. For instance, improvements in working memory and inhibitory control have been associated with better planning and execution of motor tasks (Leisman et al. 2016; Ferguson et al. 2021). This relationship suggests that cognitive training could potentially enhance motor skill development and vice versa. Additionally, the maturation of brain structures, such as the PFC, plays a significant role in the coordination and refinement of motor skills (Petrican et al. 2017).

Adolescence marks a period of continued refinement and enhancement of motor skills. During this stage, there is a notable increase in the efficiency of neural transmission due to the ongoing myelination of white matter, which supports faster and more coordinated movements (Li et al. 2024). This period also sees a reorganization of brain network interactions, particularly involving higher‐order networks, which further contributes to the improvement of motor skills. The development of motor skills during adolescence is also influenced by changes in brain connectivity. For example, there is an increase in within‐network connectivity of higher‐order networks, which supports more complex motor tasks. This increase in connectivity is part of a broader pattern of brain development that includes both conservative and disruptive modes, reflecting the strengthening of existing connections and the formation of new ones (Edde et al. 2020). Furthermore, the growth trajectories of brain network topology during childhood and adolescence do not follow a simple linear pattern. Instead, they exhibit periods of rapid development and reorganization, which are essential for the efficient functioning of motor and cognitive systems (Gozdas et al. 2019). These changes highlight the importance of a balanced approach to physical and cognitive activities to promote optimal development.

As children transition into adolescence, there is a notable increase in the density of dopamine receptors, which peaks during this period and subsequently declines with age (Wahlstrom et al. 2010; Hoops and Flores 2017). This increase in dopamine receptor density enhances the connectivity and efficiency of frontoparietal control network neural circuits, essential for cognitive control and EF. The frontoparietal control network peaks between the ages of 24 and 41 and follows an inverted U‐shaped trajectory (Li, Wang, et al. 2023), indicating that cognitive control capabilities are optimal during young adulthood.

Adolescence is also marked by significant changes in inhibitory control (Constantinidis and Luna 2019) and working memory capacity (Isbell et al. 2015). Compared to adolescence, these cognitive abilities are higher in young adulthood; working memory capacity and inhibitory control start to drop about age 30 and 35, respectively. Planning ability, another critical EF, also improves during young adulthood but declines in adults, with a small positive change in aged adults (Ferguson et al. 2021).

The relationship between physical activity and cognitive function during these stages is complex and bidirectional. Physical activity has been shown to positively influence cognitive development, enhancing brain structure and function. For instance, regular physical activity can improve the efficiency of neural circuits and support the development of cognitive control systems (Li et al. 2024). Conversely, cognitive functions such as planning and inhibitory control can influence an individual's ability to engage in and benefit from physical activity.

Additionally, the literature suggests that the external environment has the greatest impact on young and older people due to the gradual development and decline of frontally mediated EF (Craik and Bialystok 2006). This highlights the importance of providing supportive environments that promote physical activity and cognitive engagement during these critical periods of development.

#### 4.2.1 Coordination and Dexterity

Coordination and dexterity are critical components of motor skills that evolve throughout the human lifespan. These skills are essential for performing both fine and gross motor tasks, which are influenced by the intricate interplay between physical activity and cognitive function. During early development, the cerebellum plays a significant role in establishing functional hubs that are crucial for motor coordination (Kawabata et al. 2022). The removal of these hub regions from fetal network analyses has been shown to impact global efficiency, indicating their importance in functional communication during fetal life. This early establishment of coordination sets the foundation for subsequent motor skill development.

As individuals progress from childhood to adolescence, there is a notable increase in local and global connectivity within the DMN, particularly in temporal regions (López‐Vicente et al. 2021). This enhanced connectivity at age six compared to age five suggests a critical period for the development of coordination and dexterity. The maturation of these networks supports the refinement of motor skills, enabling more precise and coordinated movements.

In middle adulthood, changes in between‐network interactions become evident. There is a decrease in connectivity between higher‐order networks such as the DMN, salience network (SAL), and DAN with primary sensory networks like the motor network (MN), AUD, and visual network (VIS) (Wang et al. 2024). Conversely, there is an increase in connectivity between the DAN and SAL with the MN, AUD, and VIS, highlighting the dynamic nature of motor coordination and dexterity during this life stage (Snyder et al. 2021).

Motor skill development generally follows a U‐shaped trajectory, with peak performance observed in middle adulthood. This pattern is evident in tasks requiring planning and coordination, such as the Tower of Hanoi, where scores increase from age 10 to 30, decline from age 30 to 70, and show slight improvement thereafter (Ferguson et al. 2021). While this modest increase in late life deviates from a U‐shaped decline, it may reflect compensatory mechanisms or strategic adaptations in adults. This trajectory highlights the importance of maintaining physical activity and cognitive engagement to maintain motor skills throughout life.

An inverted U‐shaped trajectory is seen in the structural covariance between the DAN regions and areas in older people, including the motor and visual cortices as well as subcortical structures. The integrity of these structural covariance patterns peaks in middle adulthood, with reduced integrity observed in very young and very old individuals (DuPre and Spreng 2017). This decline in network integrity may contribute to the observed decrease in coordination and dexterity in older age.

The relationship between motor skills and cognitive function is further exemplified by the increased cross‐hemispheric connectivity of language‐related areas, such as Broca's and Wernicke's areas, during the first year of life. This connectivity becomes lateralized in the second year, mirroring adult patterns and supporting the development of fine motor skills involved in language production (Edde et al. 2020).

Overall, the development and maintenance of coordination and dexterity are influenced by complex interactions between various brain networks and cognitive functions. These skills are essential for daily living and are subject to changes across the lifespan, highlighting the need for a balanced approach to physical and cognitive activities to promote overall well‐being.

#### 4.2.2 Physical Activities, Neural Pruning, and Adolescent Neurocognitive Development

During early development, physical activities are essential for the maturation of motor skills (Melillo and Leisman 2010; Leisman et al. 2016). Infants and young children who actively explore their environment and engage in various forms of play tend to develop better motor coordination and cognitive abilities. As children grow, structured physical activities, such as sports, play a critical role in promoting motor and cognitive development during childhood. Activities requiring coordination, balance, and decision‐making, like soccer or gymnastics, can enhance both physical skills and EFs (Sewell et al. 2021). These benefits align with the upward trajectory of the inverted U model, as enriched motor experiences in early life contribute to stronger performance in adolescence and early adulthood. However, as demands or intensity increase, the effects may plateau or decline, emphasizing the importance of age‐appropriate and well‐balanced activity.

In adolescence, the transition to more complex and competitive sports can lead to significant improvements in cognitive control and EFs. The development of these cognitive abilities is supported by changes in neural mechanisms, which are influenced by the demands of physical activities that require quick decision‐making and problem‐solving skills (Li et al. 2024). For instance, structured training in sports like soccer has been shown to enhance creative thinking and problem‐solving abilities in young athletes, demonstrating a direct link between complex physical activity and cognitive development (Vestberg et al. 2012). This period is marked by a heightened capacity for learning and adaptation, making it an ideal time to engage in sports that challenge both the body and the mind.

Neural pruning, a critical process in adolescent brain development, involves the selective elimination of synapses. This process is essential for optimizing neural networks and enhancing cognitive efficiency. During adolescence, the brain undergoes significant structural and functional changes, which are crucial for the maturation of cognitive control and other higher‐order functions.

The concept of neural pruning is closely related to the “inverted U” relationship between movement and cognition throughout the human lifespan. This relationship suggests that both physical activity and cognitive function can influence each other, and maintaining a balance between the two is vital for overall well‐being. Neural pruning plays a significant role in this balance by refining neural circuits, thereby improving the brain's ability to process information efficiently. The distribution of inverted U‐shaped regions, which are more consistent with the frontoparietal control network, supports the idea that neural pruning is a targeted process (Li et al. 2024). This network is crucial for higher‐order cognitive functions, and its optimization through pruning can lead to improved cognitive performance. Conversely, non‐inverted U‐shaped regions are more closely related to the DAN, indicating that different neural networks may undergo distinct pruning processes.

During adolescence, there is a shift from within‐network connectivity predominance to between‐network connectivity integration. This shift is indicative of the brain's increasing ability to integrate information across different functional domains, which is facilitated by neural pruning. By eliminating redundant or less efficient synapses, the brain can enhance the connectivity between different networks, leading to more efficient information processing. The anterior‐to‐posterior gradient observed in early aging suggests that neural pruning may also follow a specific spatial pattern (Merenstein and Bennett 2022). This gradient indicates that the frontal cortex, which is involved in higher‐order cognitive functions, may undergo more extensive pruning compared to posterior regions. This pattern of pruning can help explain the changes in cognitive function observed during adolescence and early adulthood.

Furthermore, the strength of within‐network connectivity remains constant from childhood to early adolescence, after which it decreases and between‐network connectivity increases in late adolescence, highlighting the dynamic nature of neural pruning (Edde et al. 2020). This process is essential for the brain's transition from a state of specialization to one of integration, which is crucial for the development of complex cognitive abilities. In summary, neural pruning is a fundamental process in adolescent brain development that optimizes neural networks by selectively eliminating synapses. This process is closely related to the “inverted U” relationship between movement and cognition, as it enhances the brain's ability to process information efficiently. The targeted nature of neural pruning, its spatial pattern, and its role in the shift from within‐network to between‐network connectivity all contribute to the maturation of cognitive functions during adolescence (Edde et al. 2020; Li et al. 2024; Merenstein and Bennett 2022).

As individuals move into adulthood, maintaining an active lifestyle continues to be important for preserving motor skills and cognitive functions. Regular participation in physical activities has been associated with better performance in tasks that require fluid intelligence, such as processing new information and learning new skills (Sheffler et al. 2022). Moreover, the benefits of physical activity extend beyond cognitive performance to overall well‐being, reducing the risk of chronic diseases and improving mental health (Mahindru et al. 2023).

In older adults, the relationship between physical activity and cognitive function becomes even more critical. Engaging in regular exercise can help mitigate the effects of aging on the brain, promoting neuroplasticity and maintaining cognitive abilities (Liang et al. 2021; Silva et al. 2024). Studies have shown that older adults who participate in physical activities, such as walking, swimming, or yoga, exhibit better cognitive performance and slower cognitive decline compared to their sedentary peers (Li et al. 2024). Additionally, physical activity can enhance FC within the brain, which is crucial for maintaining cognitive health in later life (Edde et al. 2020).

In summary, sports and physical activities are integral to the development and maintenance of fine and gross motor skills across the lifespan. Middle childhood and adolescence are characterized by significant cognitive and motor development, influenced by the maturation of brain regions and the density of dopamine receptors. The interplay between physical activity and cognitive function during these stages underscores the importance of maintaining a balance between the two to promote overall well‐being and optimal development. From early childhood through old age, engaging in regular physical activity supports cognitive health and overall well‐being. The interplay between physical and cognitive functions underscores the importance of a balanced approach to physical activity, tailored to the needs and capacities of individuals at various stages of life (Erb et al. 2022; Li et al. 2024; Petrican et al. 2017; Schaie 2010).

### 4.3 Adult Motor Skills

Peak physical performance is a critical aspect of motor skills in adulthood, characterized by the highest levels of physical capability and efficiency. This period typically occurs in early adulthood, around the ages of 20–30 years, when individuals exhibit optimal strength, endurance, and coordination. The trajectory of physical performance follows an inverted U‐shaped curve, where performance improves during childhood and adolescence, peaks in early adulthood, and gradually declines with advancing age. The relationship between physical activity and cognitive function is further supported by the work of Gozdas et al., who performed multiple linear regressions to detect linear and quadratic developmental trajectories in motor skills (Gozdas et al. 2019). Their findings emphasize the interconnectedness of physical and cognitive development throughout the lifespan.

In adulthood, motor skills and cognitive functions are maintained through regular physical activity. Physical activity at any stage of life, but particularly in early and middle age, has been linked to a decreased risk of dementia and cognitive impairment in later life (Wang et al. 2019). This suggests that maintaining an active lifestyle can have long‐term benefits for both motor and cognitive health.

Motor skills in adulthood exhibit a complex interplay between physical activity and cognitive function, reflecting the dynamic nature of human development. During young and middle adulthood, individuals often experience a peak in motor skills (Cech and Martin 2023), which can be attributed to the optimal functioning of both cognitive and physical systems. However, as individuals transition into older adulthood, there is a noticeable decline in motor skills, which is influenced by various factors, including changes in brain structure and function, as well as motivational aspects (Cech and Martin 2023).

In young and middle adulthood, the acquisition of new skills is often driven by extrinsic motivations, such as career advancement or social recognition. This period is characterized by a high level of cognitive and physical performance, which supports the learning and refinement of complex motor skills. However, as individuals age, there is a shift towards intrinsic motivations, where personal satisfaction and the maintenance of independence become more significant drivers for engaging in physical activities and learning new skills (Sheffler et al. 2022).

As individuals age, there is a gradual decline in physical performance. This decline is influenced by several factors, including reduced muscle mass, decreased cardiovascular capacity, and changes in neuromuscular function. The study by Erb et al. (2022) indicates that age‐related slowing has a more pronounced effect on congruent trials, resulting in a decrease in the difference between congruent and incongruent response times. This finding underscores the impact of aging on motor performance and cognitive function.

The decline in motor skills during older adulthood is closely linked to changes in brain connectivity and cognitive function. Studies have indicated that there is a reorganization of brain networks, with a shift from connections between higher‐order to primary sensory networks (Malagurski et al. 2020; Deery et al. 2023). This reorganization can lead to a decrease in FC within certain brain regions, which in turn affects motor performance. For instance, older adults often exhibit a decline in within‐network connectivity for the auditory and DANs, which are crucial for coordinating complex motor tasks (Edde et al. 2020).

The concept of the inverted U‐shaped relationship between physical activity and cognitive function is well‐documented. For example, Li et al. (2024) demonstrated that young and older adults tend to be more left‐lateralized across the whole brain, indicating an inverted U‐shaped trajectory in brain laterality. This pattern suggests that both physical and cognitive functions are interrelated and follow similar developmental trajectories.

In early adulthood, individuals reach their peak physical performance, which is crucial for various motor skills. This peak is characterized by the highest levels of muscle strength, cardiovascular efficiency, and neuromuscular coordination. The efficiency of motor tasks, such as speed and accuracy, is at its maximum during this period. Petrican et al. (2017) computed an efficiency index by averaging accuracy and speed standardized scores, highlighting the importance of these factors in motor performance.

Additionally, as people age, especially in the second half of life, the effectiveness of cultural variables and resources in achieving intended results declines. This reduction in efficiency is partly due to age‐related biological changes, which impact the ability to learn and perform motor skills. Older adults may require more time and practice to achieve the same level of proficiency in motor tasks as younger individuals, highlighting the importance of tailored interventions to support motor skill development in this age group (Staudinger 2001).

Despite these challenges, some older adults maintain a high level of cognitive and motor function, often due to a combination of genetic factors, lifestyle choices, and continued engagement in physical and cognitive activities. These individuals are likely to experience a slower decline in motor skills and may even exhibit patterns of functional compensation, where other brain regions are recruited to support motor performance (Merenstein and Bennett 2022). This phenomenon underscores the potential for neuroplasticity and the ability of the brain to adapt to age‐related changes.

The relationship between motor skills and cognitive function is bidirectional. Engaging in regular physical activity can enhance cognitive function, which in turn supports better motor performance. Conversely, maintaining cognitive health through activities such as problem‐solving and memory exercises can positively impact motor skills. This interdependence highlights the importance of a holistic approach to promoting overall well‐being in adulthood and aging.

The noticeable decline in both motor and cognitive functions associated with aging is often attributed to changes in brain volume and morphometry, particularly in hippocampal and medial temporal cortical regions, which are areas associated with episodic memory and processing speed. Additionally, the accumulation of cortical iron has been linked to cognitive decline, although minimal accumulation may indicate sustained cognitive functioning in the oldest‐old individuals (Merenstein and Bennett 2022).

The relationship between motor and cognitive functions is further complicated by the differential trajectories of crystallized and fluid intelligence. Crystallized intelligence, which involves accumulated knowledge and skills, tends to be well‐maintained in older adults, whereas fluid intelligence, which involves problem‐solving and adaptability, declines with age (Craik and Bialystok 2006; Schaie 2010). This decline in fluid intelligence can impact motor skills, as tasks requiring quick adaptation and problem‐solving become more challenging.

Moreover, the loss of functional specialization in the brain, particularly in areas related to vocabulary, abstraction, and performance IQ, contributes to the decline in motor and cognitive functions in older adults (Petrican et al. 2017). This loss of specialization means that older adults may rely more on general cognitive resources rather than specialized neural networks, which can affect their ability to perform complex motor tasks. Motor skills in adulthood exhibit a complex interplay between physical activity and cognitive function, reflecting the broader dynamics of motor development across the lifespan. During adulthood, motor skills are generally well‐developed, but they are subject to gradual changes influenced by both biological aging and lifestyle factors.

The decline in motor skills observed after early adulthood can be attributed to a combination of neurobiological factors, including reductions in neural plasticity, decreased dopamine receptor density, and age‐related changes in brain connectivity that affect motor control and coordination (Wahlstrom et al. 2010; Li, Wang, et al. 2023). While some theories suggest that evolutionary selection pressures may diminish after reproductive maturity, leading to reduced optimization of motor functions (Schaie 2010), the precise mechanisms underlying this decline are still being explored. Thus, current evidence emphasizes neurophysiological changes as primary drivers of motor skill trajectories across the lifespan.

The relationship between motor skills and cognitive function is not unidirectional. Physical activity has been shown to have a positive impact on cognitive function (Leisman et al. 2016), suggesting a bidirectional relationship. Engaging in regular physical activity can help mitigate some of the age‐related declines in both motor and cognitive functions. This is supported by research indicating that maintaining flexible cognitive styles is essential for adapting to changes in life circumstances, such as retirement, and for preserving independence in older age.

Moreover, the concept of the “inverted U” relationship between movement and cognition highlights that both under‐ and over‐activity can be detrimental to overall well‐being. Optimal levels of physical activity are necessary to promote cognitive health and motor performance. This balance is crucial for adults to maintain their motor skills and cognitive functions effectively (Klimova and Dostalova 2020).

**FIGURE 5:**
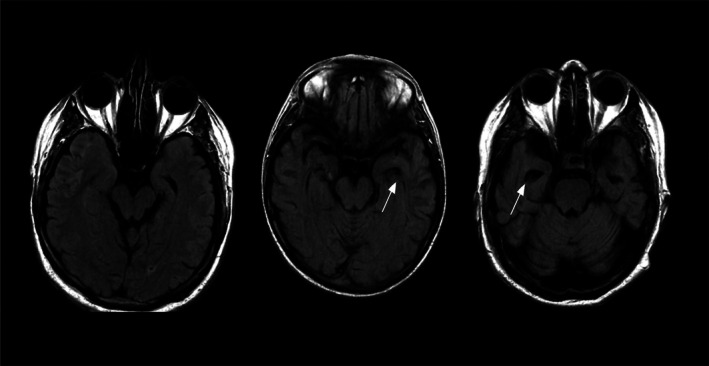
Age‐related decline as the descending limb of the inverted U. Reductions in brain volume, cortical thickness, and network connectivity in later adulthood represent the downward phase of the curve, where movement and cognition begin to deteriorate (adapted from Essig 2011, with permission).

The decline in motor skills during adulthood is also influenced by changes in brain structure and function (Cech and Martin 2023; Haywood and Getchell 2024). Changes in cognitive ability across the lifespan are associated with dissociations in the growth and deterioration of white and gray matter, as well as the functioning and maturity of particular brain regions and networks (Craik and Bialystok 2006). These neural changes can affect motor skills, as efficient motor performance relies on the integrity of these brain structures. These changes associated with normal aging are represented in Figure 5 below. Furthermore, studies have shown that different characteristics of cognitive flexibility exhibit noticeable age‐related results (Kupis et al. 2021; Varangis et al. 2022). For example, while mixing costs increase across adulthood, switch costs decrease, indicating that some cognitive processes may become more efficient with age, while others decline. This nuanced understanding of cognitive flexibility can inform strategies to maintain motor skills in adulthood.

In addition to biological factors, numerous other aspects are related to cognitive decline and, by extension, motor skills. These include genetics (Jayakody et al. 2022; Budamagunta et al. 2023), health status (Basile and Sardella 2021), physical activity (Dominguez et al. 2021; Yamasaki 2023), SES (Chan et al. 2021; Halloway et al. 2024), IQ (Quattropani et al. 2021), and physical fitness (Ferguson et al. 2021). It is important to consider the limitations of prior cohort studies of cognition, which often focused only on the level of cognitive functioning rather than developmental changes over time (Schaie 2010; Clouston et al. 2021).

In summary, peak physical performance in adulthood is a critical period characterized by optimal motor skills and cognitive function. The inverted U‐shaped trajectory of physical and cognitive performance underscores the importance of maintaining a balance between physical activity and cognitive engagement to promote overall well‐being across the lifespan. This relationship has significant implications for health and development at various life stages, highlighting the need for targeted interventions to support physical and cognitive health in both younger and older populations.

### 4.4 Motor Decline in Aging

Motor decline in aging is a multifaceted phenomenon that encompasses various aspects of physical and cognitive deterioration. As individuals age, there is a notable decline in motor functions, which can be attributed to both neurological and physiological changes. This decline is often characterized by a reduction in muscle strength, coordination, and balance, which collectively impact an individual's ability to perform daily activities (Wu et al. 2021). Cognitive control‐related brain activity follows an inverted U‐shaped trajectory, with a peak in young adulthood followed by a gradual decline with increasing age. This pattern suggests that the neural mechanisms underlying motor control and cognitive functions are closely intertwined (Leisman et al. 2016) and subject to age‐related changes (Bernard and Seidler 2014). The decline in motor functions is not uniform across all brain regions; different brain networks exhibit varying lifespan trajectories, contributing to the heterogeneity observed in motor decline (Li et al. 2024).

**FIGURE 6:**
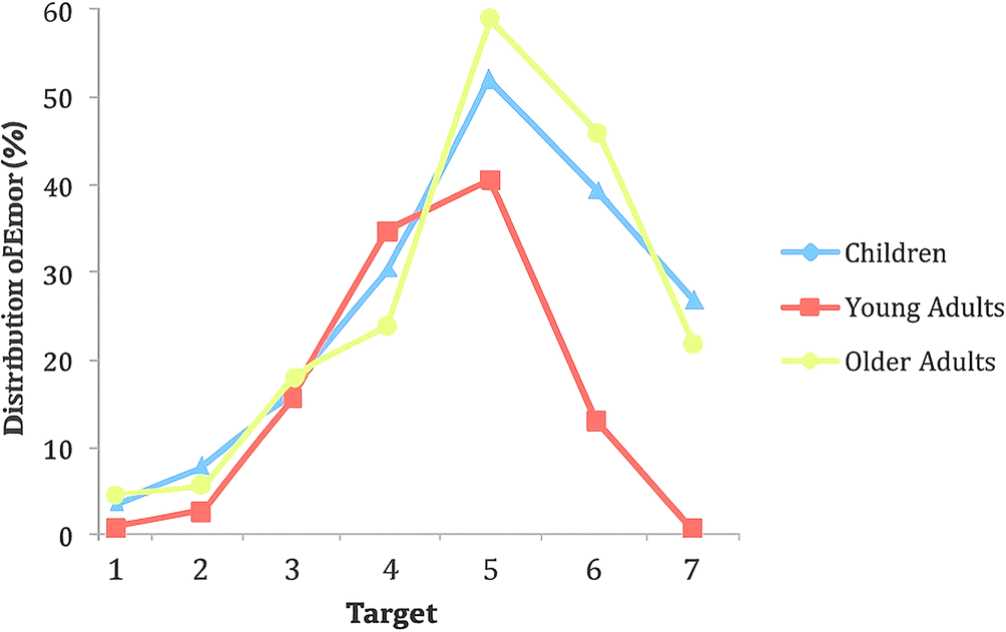
Distribution of error across targets for groups. Children and older adults perform similarly, displaying significantly greater overestimation responses in contradistinction to young adults on an imagined arm‐reaching task (adapted from Gabbard and Caçola (2011), with permission).

According to motor programming theory, an appropriate action representation of the desired activities is a crucial element of an effective outcome (Gabbard and Caçola 2011). According to Wolpert (1997), this representation is part of an internal forward model, a neuronal system that mimics the body's dynamic behavior in relation to its surroundings. According to this theory, internal models forecast (guess) how the self is mapped to external world parameters; these processes improve action planning and execution. According to Caeyenberghs et al. (2009), these representations are thought to constitute a crucial component of action planning. According to Gabbard and Caçola, motor imagery plays a role in anticipating the outcomes of one's actions. Figure 6 compares participants of various ages to show how well they can employ motor imagery to mentally depict activity across the lifespan. Responses comparing age groups with the distribution of error across goals are displayed in the figure. Children made much more mistakes on targets than adults did, and older participants made more mistakes as well, demonstrating the inverted U once more as compared to young adults.

The concept of brain reserve plays a crucial role in understanding motor decline in aging. Individuals with higher brain reserve are better able to compensate for neural degradation (Cabeza et al. 2018; Stern et al. 2019), maintaining cognitive and motor functions despite age‐related changes. Conversely, those with lower brain reserves are more susceptible to cognitive impairments and motor decline. This theory is supported by structural MRI studies, which show that individuals with high brain reserve exhibit less cognitive impairment despite similar levels of neural degradation compared to those with low brain reserve (Cabeza et al. 2018; Stern et al. 2019). Furthermore, the decline in motor functions is also influenced by the regional variation in brain aging. Studies have shown that the medial temporal regions, including the hippocampus, are particularly vulnerable to age‐related degeneration. This vulnerability is consistent across different age groups, indicating that these regions play a critical role in maintaining motor and cognitive functions. The hippocampus and entorhinal cortex, in particular, show significant age‐related volume loss, which correlates with declines in motor functions (Merenstein and Bennett 2022).

In addition to structural changes, functional changes in brain networks also contribute to motor decline. For instance, the participation coefficient of the cingulo‐opercular/salience network, which includes regions such as the insula and cingulate cortex, increases significantly from late childhood to adolescence and then stabilizes (Marek et al. 2015; Edde et al. 2020). This network is crucial for integrating sensory and motor information, and its stability in later life stages may influence the extent of motor decline (Edde et al. 2020).

The inverted U‐shaped trajectory observed in cognitive control and motor functions highlights the importance of early intervention and continuous engagement in physical and cognitive activities to mitigate the effects of aging. Engaging in regular physical activity has been shown to enhance brain plasticity and improve motor functions, thereby delaying the onset of motor decline. Additionally, cognitive training and activities that engage the brain can help maintain cognitive functions and support motor control (Staudinger 2001). Maintaining motor skills throughout adulthood is crucial for ensuring overall well‐being and functional independence. The relationship between motor skills and cognitive function is complex and dynamic, often described as an “inverted U” relationship, where both physical activity and cognitive function can influence each other positively or negatively depending on the life stage.

In older adults, maintaining motor skills becomes increasingly important as it can mitigate the decline in cognitive functions. Studies have shown that older typists, despite having slower reaction times compared to their younger counterparts, often outperform them in tasks measuring typing speed, suggesting that experience can compensate for some age‐related decline (Pauls et al. 2013). However, a contributing factor to the suboptimal phase of the inverted U trajectory is the phenomenon of functional dedifferentiation, where previously distinct neural networks begin to show overlapping activity patterns in late middle age and older adulthood (Goh 2011). This reduction in network specialization may impair the efficiency of both motor and cognitive processing. Continuous engagement in cognitively and physically demanding activities, such as bilingualism, has been shown to buffer against these declines (Craik and Bialystok 2006), emphasizing the importance of lifelong stimulation.

The neural mechanisms underlying motor skill maintenance also change with age. During early development, the brain undergoes significant maturation processes that enhance functional specialization. This maturation continues into young adulthood, where the brain's connectivity patterns stabilize. However, in late middle age and older adulthood, there is evidence of functional differentiation, where the distinctiveness of neural networks diminishes, potentially impacting both motor and cognitive functions (Petrican et al. 2017). Moreover, no matter their cognitive state, the oldest‐old adults have been shown to exhibit nonlinear white matter deterioration in the posterior and medial temporal regions. This deterioration could be an indication of accelerated natural aging, such as demyelination and cardiovascular damage, which can further complicate the maintenance of motor skills (Merenstein and Bennett 2022). Despite these challenges, it is crucial to leverage cognitive and metacognitive strategies to compensate for deficits in one area by enhancing the other (Sheffler et al. 2022).

The concept of neurocognitive scaffolding suggests that the brain adapts to aging by recruiting additional neural resources to maintain cognitive and motor functions. This adaptive mechanism highlights the importance of continuous engagement in physical and cognitive activities to support motor skill maintenance throughout adulthood (Li et al. 2024).

## 5 Integrative Perspective on Movement and Cognition Within the Inverted U Framework

A key factor in directing human goal‐directed behavior is cognitive control. Although previous research has shown that cognitive control follows an inverted U‐shaped trajectory in both behavior and anatomy, little is known about how functional brain activation changes as people age. Li, Petersen, et al. (2023) conducted a thorough meta‐analysis of 129 neuroimaging studies that included conflict tasks, including 3388 participants ranging in age from 5 to 85 years. Three key findings were reported by them: (1) The predominant pattern is an inverted U‐shaped trajectory; (2) the lifespan trajectories of brain regions related to cognitive control are heterogeneous: the DAN shows no distinct trajectories, while the frontoparietal control network follows inverted U‐shaped trajectories, peaking between 24 and 40 years; (3) both young children and the elderly exhibit greater left laterality and weaker brain activities than young to middle‐aged adults. These findings show the lifelong trajectories of cognitive control, exhibiting an inverted U trajectory and revealing diverse aging‐related changes in brain networks.

### 5.1 Early Neurocognitive Development

Early cognitive development is a critical phase in the human lifespan, characterized by rapid changes and significant growth in cognitive abilities (Melillo and Leisman 2010; Leisman et al. 2025). This period encompasses the transition from infancy through early childhood, where foundational cognitive skills are established. The development of these skills is influenced by a combination of genetic, environmental, and social factors.

During early cognitive development, children exhibit remarkable growth in various cognitive domains, including attention, memory, language, and problem‐solving abilities. Some components, such as attentional orientation, show relative stability across the lifespan, suggesting that early development in this area lays a foundational scaffold for later processes. In contrast, other cognitive functions, such as executive control or processing speed, tend to follow an inverted U‐shaped trajectory, with peaks and declines at different life stages (Li et al. 2024).

Cognitive milestones in early childhood are critical indicators of a child's developmental trajectory. During this period, the brain undergoes significant changes that lay the foundation for future cognitive abilities. From birth to around two years of age, the brain's functional networks evolve from isolated local regions to more distributed networks, a process that continues into adolescence (Edde et al. 2021). This transformation is marked by the strengthening of both short‐range and long‐range connections, which contribute to the emergence of proto networks at birth and their subsequent maturation (Edde et al. 2021; Desrosiers et al. 2024). In the first few months of life, infants exhibit rapid cognitive development. For instance, by 6 to 8 months, children show surprise reactions to changes in their environment, such as in the Switch task, where they react to all switch trials with increased looking time (Hepach 2024). This indicates an early form of cognitive processing and resource allocation. By 14 months, children only react with surprise if the changes are significant (Cesana‐Arlotti et al. 2022), suggesting a refinement in their cognitive abilities and selective attention and a change in the primary startle response reflex (Leisman et al. 2023). By 20 months, children with larger vocabularies can again show surprise to all switch trials, highlighting the interplay between language development and cognitive processing (Morse et al. 2011).

The development of visuospatial working memory is another crucial cognitive milestone in early childhood. Research indicates that brain activity related to working memory and distraction undergoes significant changes from childhood to adulthood (Li et al. 2024). These changes are essential for the development of higher‐order cognitive functions, such as problem‐solving and reasoning. Additionally, the development of FC between different brain regions is a key aspect of cognitive milestones in early childhood. For example, intrahemispheric connections between the PCC and medial PFC, as well as interhemispheric connections between bilateral PFC and motor cortices, increase with fetal age and continue to develop postnatally (Edde et al. 2020). These connections are crucial for the integration of sensory and motor information, which underpins cognitive functions such as attention, memory, and EF.

The early years are also marked by significant individual differences in cognitive development. These differences can be attributed to a variety of factors, including genetic and environmental influences. For example, the smaller but rapidly expanding intellectual repertoire causes interindividual disparities in cognitive abilities to change more quickly early in development. Greater new variance per unit of time is possible during this stage of rapid growth, which is impacted by both genetic and environmental influences (Staudinger 2001). Moreover, the complexity of cognitive tasks and the environment in which a child is raised can significantly impact cognitive development. Studies have shown that occupational cognitive complexity earlier in adulthood is associated with better white‐matter integrity and cognitive performance in midlife (Wang et al. 2019). This suggests that engaging in cognitively demanding activities during early childhood can have long‐term benefits for cognitive health.

In summary, cognitive milestones in early childhood are characterized by rapid and significant changes in brain structure and function. These changes are influenced by a combination of genetic, environmental, and experiential factors, and they lay the groundwork for future cognitive abilities. Understanding these milestones is crucial for identifying typical and atypical development and for designing interventions that can support optimal cognitive growth. Learning and memory formation are critical aspects of cognitive development in early childhood. During this period, the brain undergoes significant changes that lay the foundation for future cognitive abilities. Infants typically begin to produce their first words by the end of their first year, although they have already stored a substantial number of word forms in their memory before this age. By around 8 months, infants have likely segmented several dozen words from continuous speech and retained them in long‐term memory, even if they have not yet linked these words to specific meanings (Morse et al. 2011).

The development of cognitive control, which is essential for goal‐directed behavior, follows an inverted U‐shaped trajectory. This pattern is observed both behaviorally and anatomically, with brain activation associated with cognitive control also following this trajectory, highlighting the dynamic nature of brain development across the lifespan. Early infancy is when the gray matter volumes in the frontal and parietal regions, which are essential for cognitive control tasks, rise, and early old age is when they start to atrophy. The distinct paths of different brain subregions demonstrate the hierarchical patterns of brain development and degeneration. These patterns are crucial for understanding the complexities of cognitive development and the factors that influence it. For example, throughout life, the anterior and posterior hippocampus experience changes in macro‐ and micro‐structure that are associated with memory function. Longitudinal studies have shown that these changes are significant and can impact memory function at different life stages (Merenstein and Bennett 2022).

Moreover, cognitive performance is influenced by both biological and cultural systems. Age disparities in proficient chess play, for instance, show how these variables can influence cognitive capacities. The average age of 46 years is the age at which a world championship is first won in correspondence chess, when players have more time to think things through, as opposed to roughly 30 years of age in tournament chess, where players must make decisions more quickly (Staudinger 2001). This example underscores the importance of considering both innate and environmental influences on cognitive development.

The relationship between cognitive control and brain activation is further supported by the observation that the distribution of brain activation is more asymmetrical in young and old people than in young to middle‐aged adults. This asymmetry may reflect the hierarchical nature of brain development in different regions, with certain areas maturing earlier and others later (Leisman et al. 2022; Li et al. 2024). Li et al. (2024) indicate that understanding these trajectories is necessary for developing targeted interventions supporting cognitive health across the lifespan.

The interaction between physical activity and cognitive development is particularly noteworthy. Engaging in physical activities has been shown to enhance cognitive functions, such as executive functioning and memory. For instance, performance in tasks requiring planning and executive functioning, like the Tower of Hanoi, is associated with higher levels of fluid ability and spatial memory (Salthouse and Davis 2006). These findings underscore the importance of physical activity in promoting cognitive development during early childhood.

Moreover, the influence of social and educational environments cannot be overstated. Children who are exposed to rich social networks and high levels of education tend to exhibit better cognitive outcomes, even in the presence of brain degenerative pathologies later in life (Nie et al. 2021; Li et al. 2021). This highlights the role of early educational interventions and social interactions in shaping cognitive trajectories.

The concept of the inverted U‐shaped relationship between cognitive development and age is also evident in early childhood. Studies have shown that cognitive performance improves from childhood to early adulthood, followed by a decline in later years (Erb et al. 2022). This pattern suggests that early cognitive development is a period of significant growth, setting the stage for peak cognitive performance in young adulthood.

### 5.2 Problem‐Solving Abilities Across the Lifespan

Problem‐solving abilities in early childhood are a critical aspect of cognitive development, reflecting the child's capacity to navigate and resolve challenges through various strategies. These abilities are closely linked to working memory and EF, which facilitate the planning and execution of tasks. Research indicates that individuals with higher working memory capacity tend to exhibit superior planning abilities, a relationship that persists regardless of age (Braver and West 2011; Kirova et al. 2015). This suggests that the cognitive mechanisms underlying problem‐solving are robust and can be observed across different developmental stages. Still, as we shall see, specific changes are visible as an inverted U.

Working memory plays a crucial role in problem‐solving by allowing children to hold and manipulate information while formulating and executing plans. Traditional tower tasks, for example, which are frequently used to evaluate planning skills, call for the inhibition of prepotent responses and the formulation of ideas for problem‐solving. These tasks illustrate how working memory supports the ability to think ahead and sequence steps to achieve a goal (Ferguson et al. 2021).

The development of problem‐solving skills is also influenced by the brain's evolving structure and function. Studies have shown that cognitive control brain activities follow an inverted U‐shaped trajectory, with peak performance occurring between the ages of 24 and 41 years (Li et al. 2024). This pattern reflects the maturation and subsequent decline of cognitive control networks, which are essential for effective problem‐solving. The frontoparietal control network, in particular, is associated with higher‐order cognitive processes, including planning and decision‐making.

Moreover, the relation between EF and age, such as task switching and mixing costs, highlights the dynamic nature of cognitive development. Individuals with greater switch costs tend to have smaller mixing costs, indicating a trade‐off between different aspects of EF (Ferguson et al. 2021). This interplay suggests that as children develop, they refine their problem‐solving strategies, balancing the demands of different cognitive tasks.

Interestingly, the link between superior cognitive function and changes in brain connectivity further underscores the importance of neural development in problem‐solving. Greater expression of within‐network connectivity profiles has been associated with better working memory performance, suggesting that efficient neural communication supports cognitive abilities (Petrican et al. 2017). This supports the idea that early childhood EF development depends on a transition from bottom‐up to top‐down regulatory mechanisms.

In addition to neural factors, environmental and experiential influences play a significant role in shaping problem‐solving abilities. Proficient learners can discern relevant information and anticipate future steps based on their extensive knowledge and experience (Sheffler et al. 2022). This expertise allows them to respond effectively to various challenges, demonstrating the importance of practice and exposure to diverse problem‐solving scenarios.

### 5.3 Social Cognition in Early Cognitive Development

A crucial component of early childhood cognitive development, social cognition includes the capacity to comprehend and communicate with others. This includes recognizing emotions, understanding social cues, and developing empathy. The development of social cognition is influenced by various factors, including brain maturation, environmental interactions, and individual experiences. The human brain undergoes significant topological modifications from birth through adolescence, which are influenced by cognitive abilities and sex. Social cognition, which includes the capacity to comprehend and communicate with others, is a crucial component of early childhood cognitive development. The establishment of small‐world properties in functional brain networks early in development supports efficient information processing, which is essential for social cognition (Gozdas et al. 2019).

The trajectory of social cognition development can vary significantly among individuals due to differences in methodologies and processing requirements (Zimmermann and Meier 2006). These variations highlight the importance of considering individual differences when studying social cognition. Additionally, working memory, cognitive flexibility, inhibitory control, and other cognitive processes are all linked to the development of social cognition (Proverbio 2023; Stangl et al. 2023). These functions continue to develop over adolescence and into early adulthood, contributing to the overall maturation of social cognition (Ferguson et al. 2021).

The concept of the “inverted U” relationship between movement and cognition is also relevant to social cognition. It has been demonstrated that physical activity affects cognitive performance, including social cognition, by promoting brain health and enhancing neural connectivity. This relationship underscores the importance of maintaining a balance between physical activity and cognitive engagement to support optimal social‐cognitive development throughout the lifespan (Li et al. 2024). Furthermore, socialization events, both normative and idiosyncratic, play a significant role in shaping social cognition. These events, such as formal schooling and mentoring, provide opportunities for individuals to acquire and refine social cognitive skills. The knowledge gained from these experiences is represented both internally, through semantic networks, and externally, through various forms of media (Staudinger 2001).

As individuals age, the brain‐behavior associations related to social cognition may attenuate, particularly in middle and older adulthood. This attenuation can affect various aspects of cognitive function, including episodic memory, verbal reasoning, and sensorimotor processing (Petrican et al. 2017). Understanding these changes is crucial for developing interventions that can support social cognitive function across the lifespan. In summary, the development of social cognition in early childhood is a complex process influenced by brain maturation, individual experiences, and environmental interactions. The interplay between physical activity and cognitive function further emphasizes the need for a holistic approach to promoting social cognitive development. By considering these factors, we can better understand the mechanisms underlying social cognition and develop strategies to support its development throughout the human lifespan.

### 5.4 Language Acquisition in Early Childhood

Language acquisition during early childhood serves as a foundation not only for cognitive development but also for later social cognition. As linguistic skills mature, they enable children to interpret and engage with others' mental states more effectively, facilitating the development of social understanding.

Piaget's theory of cognitive development (Piaget 1978) provides a framework for understanding how children acquire language. According to this theory, children's cognitive abilities evolve through a series of stages, beginning with sensorimotor responses and progressing to more abstract representations. Initially, children's language skills are closely tied to their sensory and motor experiences. As they grow, these skills become more sophisticated and symbolic, allowing for the development of complex language structures (Craik and Bialystok 2006). We will examine Piaget's theories of cognitive development in the context of the inverted U over the lifetime.

The relationship between language acquisition and cognitive development is further illustrated by the functional coupling patterns of brain networks. Research has shown that the DMN and the Auditory/Language network exhibit age‐related differences in their connectivity patterns (Skeide and Friederici 2016; Hertrich et al. 2020). The DAN, which is linked to goal‐directed attention, has a stronger negative relationship with the DMN than it does with the subcortical network as people age (Sato et al. 2014; Rebello et al. 2018; Onofrj et al. 2022). These changes in network connectivity reflect the dynamic nature of language processing and its integration with other cognitive functions. Moreover, the cerebral resources that are most flexible and change throughout a person's life can be inferred from the developmental trajectory of connections within brain networks. This adaptability is crucial for language acquisition, as it allows children to learn and refine their language skills through interaction with their environment. The malleability of these neural resources also makes them vulnerable to decline with aging, highlighting the importance of early language development for long‐term cognitive health (Petrican et al. 2017).

Educational attainment and cognitive abilities in childhood, such as intelligence quotient (IQ) and bilingualism, have been shown to influence language acquisition and cognitive development. Higher levels of education and cognitive abilities are linked to a lower risk of dementia and cognitive impairment in later life. This suggests that promoting language development and cognitive skills in early childhood can have long‐lasting benefits for overall cognitive health (Wang et al. 2019).

The process of language acquisition is also influenced by the configuration of brain hubs, which gradually change to an adult‐like topology during the first years of life. These changes in brain network topology are associated with the maturation of functional brain networks, which support the development of language and other cognitive abilities. Understanding the developmental trajectories of these networks can provide valuable insights into the neural underpinnings of language acquisition and cognitive variability in children (Edde et al. 2020).

### 5.5 Neurocognitive Development in Middle Childhood

Significant alterations in the structure and function of the brain are hallmarks of middle childhood cognitive development, which is essential for improving cognitive capacities. The frontal and parietal gray matter volumes, which are crucial for cognitive control tasks, exhibit significant increases during this time (Wilke et al. 2007; Ando et al. 2021). Higher order cognitive functions including planning, problem‐solving, and attention are mediated by these areas.

The development of cognitive functions during middle childhood is also influenced by environmental factors. For instance, children who grow up in environments rich in cognitively stimulating resources, such as books and musical instruments, tend to show increased intrinsic motivation for learning. This is particularly evident in children from higher SES backgrounds, who generally have better access to such resources compared to their lower SES counterparts (Sheffler et al. 2022). Furthermore, the transition from early to middle childhood involves changes in brain connectivity patterns. Studies have shown that during this period, there is a significant development in the structural covariance networks, which are crucial for efficient cognitive functioning. These networks undergo substantial reorganization, which supports the improvement of cognitive abilities such as working memory and EF (DuPre and Spreng 2017).

The cognitive development trajectory during middle childhood can be described by an inverted U‐shaped pattern, where cognitive abilities improve significantly before reaching a plateau and eventually declining in later stages of life. This pattern is observed in various cognitive control‐related brain regions, indicating that the development of these regions follows a similar trajectory (Li et al. 2024).

Additionally, cognitive aging research highlights that the cognitive development observed in middle childhood sets the foundation for future cognitive health. Individuals who achieve higher levels of cognitive functioning during this period are more likely to maintain cognitive abilities into older age, even if they become physically frail (Schaie 2010). This underscores the importance of fostering cognitive development during middle childhood to promote long‐term cognitive health.

### 5.6 Adolescent Neurocognitive Development

Adolescent brain changes are characterized by significant structural and functional transformations that have profound implications for cognitive and behavioral development. During adolescence, the brain undergoes extensive remodeling, particularly in regions associated with EF, such as the PFC. This period is marked by the continued myelination of neural pathways, which enhances the efficiency of neural transmission and supports the maturation of cognitive control and EF.

One concrete example of an environmental factor influencing both motor and cognitive performance is blue light exposure. Recent findings demonstrate that evening exposure to blue light, particularly after 9:00 p.m., can negatively affect sleep quality, cognitive performance, and motor function the next day (Souissi et al. 2020). This highlights how everyday environmental conditions can modulate brain functioning and contribute to deviations in the inverted U‐shaped trajectory.

The PFC, which is crucial for decision‐making, planning, and social behavior, continues to develop well into early adulthood. This ongoing development is associated with improvements in working memory and attention, as well as the ability to regulate emotions and behaviors. The dorsolateral PFC, in particular, shows significant changes during adolescence, which are linked to the maturation of cognitive control and EF (Edde et al. 2020).

In addition to structural changes, there are notable shifts in brain connectivity during adolescence. The connectivity within the DMN, which includes regions such as the medial PFC, posterior cingulate, and precuneus, shows age‐related differences (Sherman et al. 2014; Washington and VanMeter 2015). Older adults tend to have lower connectivity between these regions compared to younger individuals, indicating that the brain's functional architecture continues to evolve throughout the lifespan.

The concept of brain maintenance suggests that age‐related brain alterations are limited in those who have cognitive capacities comparable to those of younger adults. This theory posits that maintaining a young‐like brain is associated with a relative lack of brain pathology and is comparable to the term “resistance” used in Alzheimer's disease research (Merenstein and Bennett 2022). This idea underscores the importance of cognitive and physical activities in promoting brain health and mitigating the effects of aging. Furthermore, activation studies have shown that age‐related neuronal dedifferentiation may be related to the breakdown of brain‐behavior linkages found in older adults (Petrican et al. 2017). This phenomenon highlights the dynamic nature of brain development and the interplay between structural and functional changes across the lifespan.

In summary, the adolescent brain undergoes significant changes that are crucial for the development of cognitive control and EF. These changes are characterized by continued myelination, shifts in brain connectivity, and the maturation of the PFC. The concept of brain maintenance further emphasizes the importance of maintaining cognitive and physical activities to promote brain health throughout the lifespan. These insights have important implications for supporting healthy development and addressing the challenges associated with adolescence.

Adolescent cognitive development is a critical phase characterized by significant changes in brain structure and function, which influence various cognitive abilities. During this period, the brain undergoes extensive remodeling, particularly in the PFC, which is associated with higher‐order cognitive processes such as planning, decision‐making, and impulse control (Li et al. 2024). This developmental stage is marked by a transition from more concrete to abstract thinking, enabling adolescents to engage in more complex problem‐solving and reasoning tasks.

The development of EF also follows a distinct trajectory during this period. From adolescence to early adulthood, EF improves developmentally. After that, it declines throughout adulthood, with the decline becoming more pronounced as people age (Ferguson et al. 2021). This pattern underscores the importance of early interventions and continuous support to maintain cognitive functions throughout life.

Changes in adolescent EF include cognitive processes such as inhibitory control, cognitive flexibility and working memory. These functions are essential for academic success and everyday decision‐making. Research indicates that EF and fluid intelligence are strongly related, suggesting that improvements in one area can positively influence the other (Salthouse and Davis 2006). This interrelation underscores the importance of fostering environments that support both physical and cognitive activities to promote overall well‐being during adolescence.

Moreover, the development of cognitive abilities during adolescence is influenced by various factors, including social interactions, educational experiences, and environmental stimuli. Social processes, such as peer relationships and family dynamics, play a significant role in shaping cognitive development by providing opportunities for learning and emotional support (Schaie 2008, 2010). Educational settings that challenge students intellectually and encourage critical thinking can further enhance cognitive growth.

The hierarchical development trajectories in different brain networks also highlight the complexity of cognitive development during adolescence. For example, the DAN, which is involved in attentional orientation, shows a relatively stable pattern, while the frontoparietal control network, associated with EF, follows an inverted U‐shaped trajectory (Li et al. 2024). This dissociation suggests that different cognitive functions may develop at varying rates and are influenced by distinct neural mechanisms.

Moreover, the reorganization of network interactions in the brain during young to middle adulthood suggests that higher‐order networks undergo significant changes. This reorganization includes an increase in connectivity between primary sensory and high‐order networks (Edde et al. 2020). These changes highlight the dynamic nature of cognitive development and the need for adaptive learning strategies.

From infancy to early adulthood, fluid intelligence, which includes the capacity for reasoning and problem‐solving, generally increases Mitchell et al. (2023) and then decreases from young/middle adulthood to older adulthood (Mitchell et al. 2023). On the other hand, throughout adulthood, crystallized intelligence, based on acquired knowledge and experience, continues to rise, albeit at a slower rate (Scherrer et al. 2024). This distinction between fluid and crystallized intelligence emphasizes the need for different educational approaches at various life stages.

Vicarious learning, or learning through observation and modeling, is another important aspect of social and emotional learning. Children learn new skills not only through formal instruction but also by observing and imitating the actions of those around them (Sheffler et al. 2022). This form of learning is particularly effective in early childhood and continues to play a role in cognitive development throughout life.

The concept of the “inverted U” relationship between movement and cognition is particularly relevant during adolescence. This relationship suggests that both physical activity and cognitive function follow a trajectory where moderate levels of activity and engagement are associated with optimal cognitive performance, while too little or too much can be detrimental (Pauls et al. 2013). For instance, physical activity has been shown to enhance cognitive function by promoting neurogenesis, synaptic plasticity, and vascularization in the brain, which are crucial for learning and memory (Wang et al. 2019). Conversely, excessive physical activity without adequate rest can lead to cognitive fatigue and impairments.

#### 5.6.1 Adolescent Risk‐Taking Behaviors

Risk‐taking behaviors during adolescence are a significant area of interest in understanding the developmental changes in the brain and their implications. Adolescence is characterized by a heightened propensity for risk‐taking, which can be attributed to the ongoing maturation of brain regions involved in EF and cognitive control. The PFC, which is crucial for decision‐making and impulse control, continues to develop well into early adulthood. This delayed maturation can result in an imbalance between the PFC and the limbic system, which is associated with emotions and reward processing (Ferguson et al. 2021; Li et al. 2024; Merenstein and Bennett 2022).

While it is known that the brain undergoes structural and functional changes during adolescence, the relationship between brain network architecture and cognitive capacities during development has not been well investigated, but it is known that structural and functional changes in the brain occur throughout adolescence. These changes can influence behaviors, including risk‐taking. The idea that cognitive control abilities are not fully formed in children and may deteriorate in the elderly is supported by the frontoparietal control network, which is more active in young adults than in children and the elderly (Li et al. 2024). This suggests that adolescents, who are in a transitional phase, may exhibit varying levels of cognitive control, contributing to their risk‐taking behaviors.

Furthermore, the development of EFs such as working memory, planning, cognitive flexibility, and inhibitory control, which are essential for regulating behavior and making informed decisions, shows significant changes during adolescence. These EFs are crucial for managing risk‐taking behaviors, and their development can be influenced by both biological and environmental factors (Ferguson et al. 2021). The inclusion of adolescents in studies of EFs provides a comprehensive understanding of the distinct developmental trajectories and highlights the importance of this age group in research.

The concept of the “inverted U” relationship between movement and cognition throughout the human lifespan also plays a role in understanding risk‐taking behaviors. Physical activity has been shown to influence cognitive function, and vice versa, suggesting that a balance between the two is essential for overall well‐being. During adolescence, engaging in physical activities can promote cognitive development and potentially mitigate risk‐taking behaviors by enhancing EF and cognitive control (Craik and Bialystok 2006).

Additionally, significant effects have been noted when brain network architecture interacts with cognitive capacities during development. For instance, there is a significant relationship between age throughout development and the structure of the brain's functional networks, indicating that the brain's structural and functional organization evolves (Gozdas et al. 2019). This evolution can impact behaviors, including risk‐taking, as adolescents navigate the complexities of their developing brains.

In summary, risk‐taking behaviors during adolescence are influenced by the ongoing maturation of brain regions involved in EF and cognitive control. The delayed development of the PFC, the interaction between brain network topology and cognitive abilities, and the balance between physical activity and cognitive function all contribute to the propensity for risk‐taking in this age group.

### 5.7 Identity Formation

**FIGURE 7:**
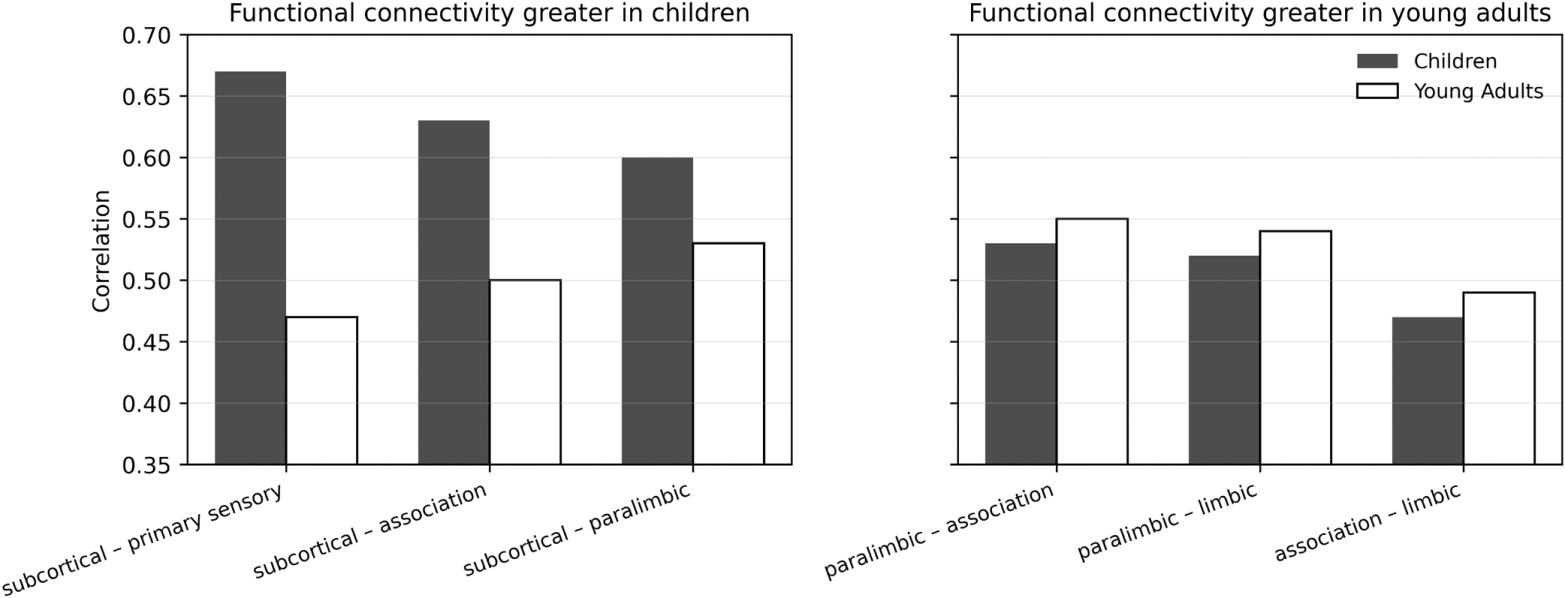
Large‐scale brain networks in children and adolescents are characterized, arranged, and developed using graph‐theoretical metrics.

The adolescent brain undergoes extensive remodeling, particularly in regions associated with EFs functions, such as the PFC. This area is responsible for higher‐order cognitive processes, including decision‐making, impulse control, and social behavior. The frontal lobe hypothesis suggests that these changes disproportionately affect the anterior brain regions, leading to variations in cognitive performance and behavior (Merenstein and Bennett 2022). As adolescents navigate the complexities of identity formation, the maturation of these brain regions supports their ability to integrate personal experiences and social feedback into a coherent self‐concept. Functional connectivity within the brain also evolves during adolescence. Studies have shown that there is a decrease in within‐network connectivity, which is indicative of a more integrated and efficient brain network organization. This reorganization is essential for the development of complex cognitive abilities and the capacity to form a stable identity. The changes in FC highlight the dynamic nature of the adolescent brain and its adaptability in response to environmental and social influences. Figure 7 below illustrates quite clearly the shift in functional connectivities between early childhood and adolescence, where in early childhood there exist greater local connectivities between brain regions, and adolescents demonstrate significantly greater global connectivities (Mills et al. 2016). The aged are best represented with a return to local connectivities (Lopez‐Larson et al. 2011; Damoiseaux 2017).

In a comprehensive review, Sala‐Llonch et al. (2015) pointed out that healthy aged individuals exhibit lower FC than young adults. The default mode network appears to be a severely degraded system in healthy aging, and this decreased connectivity impacts the primary brain networks and explains age‐related cognitive changes (Song et al. 2014). It is in some way as if we return to an early stage of brain organization in advanced aging (Fair et al. 2009).

Moreover, the localization of functional regions in the brain shifts with age, reflecting the ongoing development and specialization of neural circuits (Edde et al. 2020). These changes are crucial for the refinement of cognitive and emotional processes that underpin identity formation. The ability to process and respond to social information becomes more sophisticated, enabling adolescents to better understand themselves and their place in the world.

The interplay between cognitive control and emotional regulation is another important aspect of identity formation. As adolescents develop, their neural systems supporting these functions undergo significant changes. Understanding how these systems evolve can provide insights into the mechanisms underlying identity development and the challenges that may arise during this period (Li et al. 2024). The balance between cognitive and emotional processes is essential for the successful navigation of identity‐related tasks and the establishment of a stable self‐concept.

Sociocultural factors also play a significant role in identity formation. Accumulated cultural resources, such as educational level and occupational status, influence the timing and nature of identity development (Schaie 2010). These factors interact with neurobiological changes to shape the trajectory of identity formation, highlighting the importance of considering both internal and external influences in understanding this complex process.

In summary, identity formation during adolescence is a multifaceted process driven by neurobiological changes and sociocultural influences. The maturation of brain regions involved in EF, the reorganization of FC, and the interplay between cognitive and emotional processes all contribute to the development of a coherent self‐concept. Understanding these factors can provide valuable insights into the mechanisms underlying identity formation and the challenges that adolescents may face during this critical development period.

### 5.8 Adult Neurocognitive Function

Cognitive functions in adulthood exhibit a complex interplay of various factors, including brain structure, cognitive reserve, and lifestyle influences. Throughout life, the brain's cognitive control areas follow an inverted U shape, with each region experiencing peak activity at a different age. This suggests that cognitive control does not develop uniformly across all brain regions (Li et al. 2024). Adult cognitive function is influenced by various factors, including physical activity, brain‐behavior relationships, and age‐related changes in brain function. Research indicates that physical activity at any stage of life, particularly during early and middle age, is associated with a reduced risk of cognitive impairment and dementia in later years (Wang et al. 2019). This suggests that maintaining an active lifestyle can have long‐term benefits for adulthood and aging, represent critical necessities in the lifespan where the interplay between movement and cognition undergoes significant transformations. During young adulthood, cognitive control abilities are at their peak, exemplified by elevated functional brain activation, which is associated with better performance on activities requiring cognitive control. This period is marked by the most effective cognitive control system, suggesting that young adults exhibit optimal brain activity and cognitive function (Rieck et al. 2021).

Research has shown that EF abilities exhibit significant changes across the lifespan. For instance, he oldest‐old show less activity in the dorsolateral prefrontal and posterior parietal cortices than younger people, despite both groups having comparable high accuracy levels on an executive control test (Merenstein and Bennett 2022). This suggests that although older adults can maintain performance levels, they may rely on different neural mechanisms compared to younger individuals.

The development and decline of EF follow an inverted U‐shaped trajectory, with peak performance typically occurring in early adulthood. This pattern indicates that EF abilities improve during childhood and adolescence, reach their zenith in young adulthood, and gradually decline with advancing age (Li et al. 2024). The heterogeneity in lifespan trajectories across different brain regions further underscores the complexity of EF development and aging.

EF assessment tasks need to be sensitive enough to identify age‐related reductions and take general cognitive processes into consideration. In a study that adjusted for response latencies, IQ, and SES, four EF tasks were found to be age‐sensitive, providing a comprehensive description of EFs across the lifespan (Ferguson et al. 2021). This highlights the importance of considering various factors that can influence EF performance when evaluating age‐related changes. Moreover, early declines in EF have been observed in middle‐aged adults, with some studies reporting a linear decline across adulthood that becomes steeper after the age of 65 (Ferguson et al. 2021). This early onset of decline emphasizes the need for interventions aimed at maintaining EF abilities throughout adulthood.

The relationship between movement and cognition is also crucial for understanding EF. In older adults, regular physical activity has been linked to improved cognitive function and a lower risk of cognitive deterioration (Wang et al. 2019). This interaction between mental and physical health emphasizes how crucial a balanced lifestyle is for fostering general wellbeing. As individuals transition into middle age, there is a notable shift in cognitive flexibility. Studies indicate that while switch costs decrease, mixing costs increase, implying that middle‐aged adults face challenges in maintaining task sets, whereas younger adults struggle more with switching between tasks (Ferguson et al. 2021). This shift highlights the nuanced changes in EFs that occur during adulthood, which have often been overlooked in favor of studying childhood development or comparing young and older adults.

In older adulthood, cognitive control abilities begin to decline. It has been demonstrated that when compared to younger individuals, older adults frequently show less brain activity in the frontoparietal regions, associated with a decline in functional capabilities (Koshino et al. 2023). This reduction in brain activity is associated with decreased performance on cognitive control tasks, reflecting the broader trend of cognitive decline with aging. Neuropathological processes in the PFC, decreased medial temporal lobe volume, and decreased hippocampus activation have all been linked to an accelerated decline in EF and daily functioning in older age, especially in patients with dementia (Hedden and Gabrieli 2004). Furthermore, frontal theta oscillations during cognitive function assessment (e.g., working memory task) indicate that a decrease in event‐related frontal theta activity (acquired via EEG) occurs in progressive MCI patients versus stable‐MCI patients and could be utilized as a biomarker of rapid cognitive decline in older people reporting pre‐dementia cognitive impairments associated with working memory (Deiber et al. 2009). Furthermore, compared to age‐matched controls, persons with a range of age‐related neurological illnesses (i.e., dementia patients) had higher resting theta power and lower resting alpha power (Klimesch et al. 1999; Deiber et al. 2009). Therefore, an increase in theta and a decrease in alpha power during rest are characteristics of the EEG in individuals with dementia. Interestingly, alpha reactivity (during a cognitive challenge) tends to decrease with age.

The relationship between physical activity and cognitive function also evolves with age. While single‐component physical activities, such as aerobic exercises, have not been conclusively shown to prevent cognitive decline or dementia in older adults, multidomain interventions that combine various types of physical and cognitive activities may help delay cognitive decline (Castro et al. 2025). This suggests that a holistic approach to physical activity, incorporating multiple domains, could be more effective in maintaining cognitive health in older adults. Additionally, social interaction is essential for maintaining cognitive health as individuals age. Evidence indicates that a higher incidence of dementia in later life is linked to social disengagement, weak social networks, and social isolation (Wang et al. 2023). A consistent framework for assessing brain activity across age groups is provided by conflict processing, a subdomain of cognitive control. This consistency makes it a valuable tool for cross‐age comparisons, although caution is necessary when generalizing findings to other subdomains of cognitive control (Li et al. 2024). Future research should aim to develop more precise models for comparing cognitive control across the lifespan, recognizing the heterogeneity in brain network trajectories.

**FIGURE 8:**
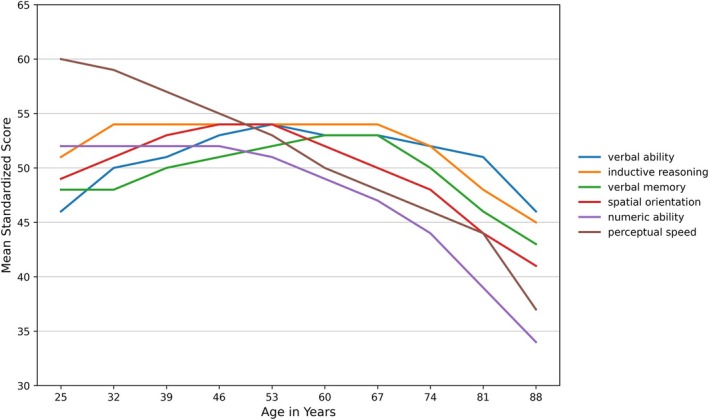
Data from the Seattle Longitudinal Study, which monitored, between 1956 and 2012, individuals' cognitive capacities. The findings demonstrated that middle‐aged adults outperform young adults on four of six cognitive tasks. Up to the 70s, verbal memory, spatial abilities, vocabulary, and inductive reasoning—the ability to conclude from specific examples—all improve with age (Schaie et al. 2005). Perceptual speed and numerical computation, however, deteriorate in middle and late adulthood.

**FIGURE 9:**
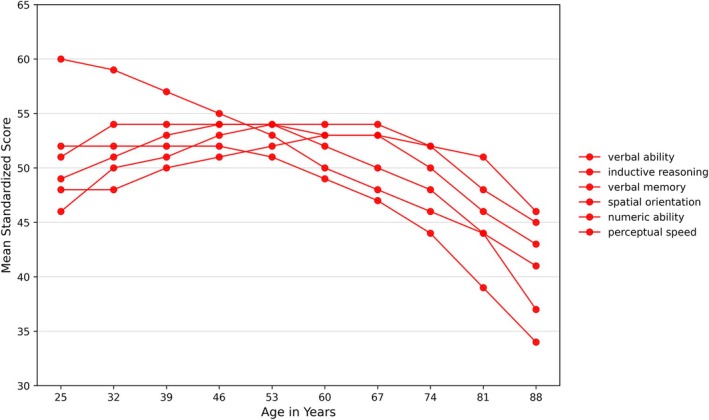
Cross‐sectional age patterns are illustrated in: (a) fluid thinking (Tucker‐Drob 2019), (b) crystallized knowledge, and (b) the prevalence of dementia based on performance on the Woodcock–Johnson Tests of Cognitive Abilities, Third Edition (Woodcock et al. 2001). The Medical Research Council Cognitive Function and Aging Study II (*N* = 7720); Matthews et al. (2013) provided the prevalence rate data for dementia.

An inverted U‐shaped trajectory is evidenced in brain activity associated with cognitive control, with peak performance in young adulthood and a decline in older age (Li et al. 2024). Figures 8 and 9 present lifespan data on cognitive abilities derived from longitudinal and cross‐sectional studies, including the Seattle Longitudinal Study and assessments of fluid and crystallized intelligence. These data reveal heterogeneous patterns: while certain capacities such as processing speed and working memory demonstrate clear inverted‐U trajectories, others, including crystallized intelligence, show more linear or plateaued improvements well into older adulthood. Conversely, some abilities like fluid reasoning exhibit continuous decline after early adulthood without a pronounced peak. These discrepancies underscore that the inverted U is not a universal template but rather a guiding heuristic that applies selectively across cognitive domains. Such variability likely reflects differences in neurobiological substrates, task demands, compensatory mechanisms, and environmental influences. Accordingly, our framework is best interpreted as a domain‐sensitive model, where certain processes (e.g., EF, motor coordination) more consistently exhibit non‐linear trajectories than others.

**FIGURE 10:**
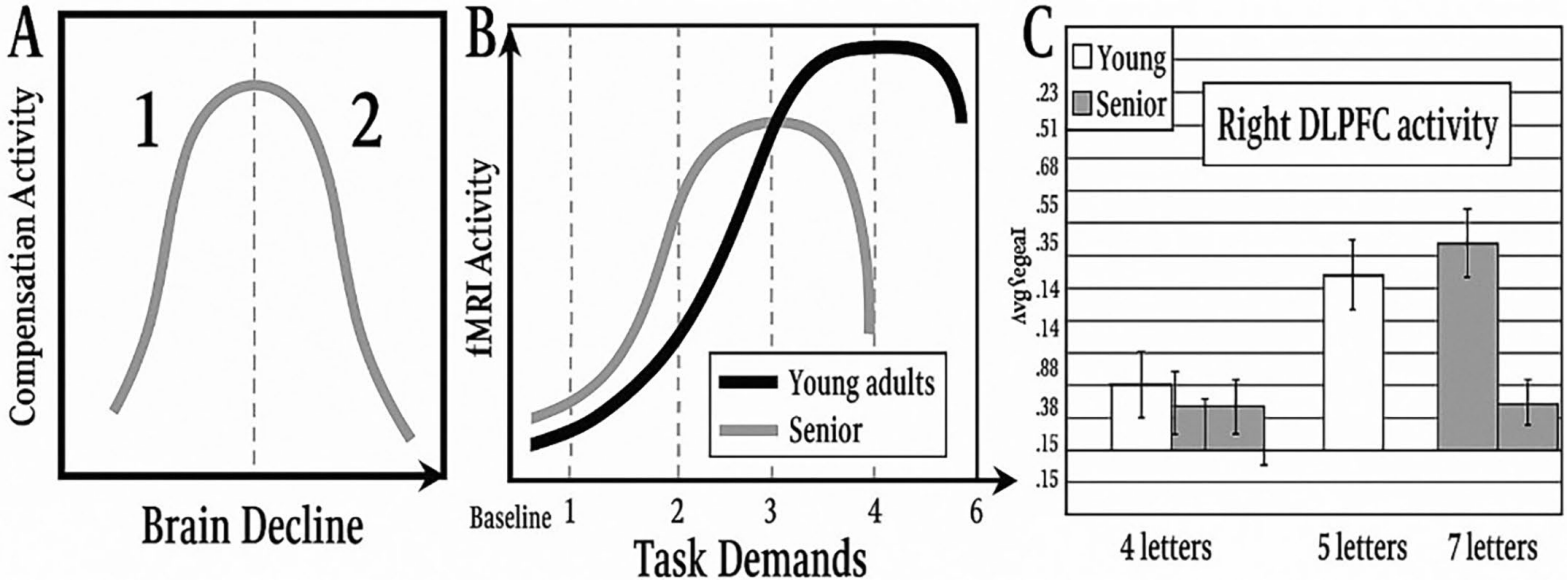
dlPFC activity tends to increase in tandem with task demands (C), possibly reflecting resource recruitment, but eventually declines, possibly signaling capacity limitations. The mediotemporal region (MTL) (hippocampus) and posterior cortical regions (parietal and retrosplenial cortices) are less connected in older people, although the entorhinal cortex, another MTL region, and bilateral dorsolateral PFC regions are significantly more connected. Older individuals tend to have a leftward shift in the inverted‐U function (A). The amygdala is equally engaged in both age groups during successful emotional encoding (B).

In further support of developmental cognitive trajectories having an inverted‐U relationship with brain development and decline, Figures 9 and 10, respectively, illustrate that notion (Figure 9B). Evidence that PFC activity tends to increase in tandem with task demands, possibly reflecting resource recruitment, but eventually declines, possibly signaling capacity limitations, supports this criterion (Rypma and D'Esposito 2000; Mattay et al. 2006; Schneider‐Garces et al. 2010; Cappell et al. 2010). Reuter‐Lorenz and Cappell (2008) noted that older individuals tend to have a leftward shift in the inverted‐U function (Figure 9B). This shift can be read as evidence that they use more brain resources to meet lower task demands, leaving less for higher task demands. In support, recent findings suggest that insufficient DLPFC top‐down online‐processing‐resources remain to activate or inhibit verbal WM neural networks in interference‐rich conditions or across WM retention intervals. This could be the cause of excessive alpha activity under left prefrontal electrodes in older adults (Meiron et al. 2021). These findings in older healthy adults are different from earlier younger controls' findings displaying bilateral parietal‐prefrontal alpha dynamics that are functionally relevant during WM storage periods, unlike lateralized alpha activity (lateralized functional *alpha inhibition*) observed in older people during WM retention intervals.

**FIGURE 11:**
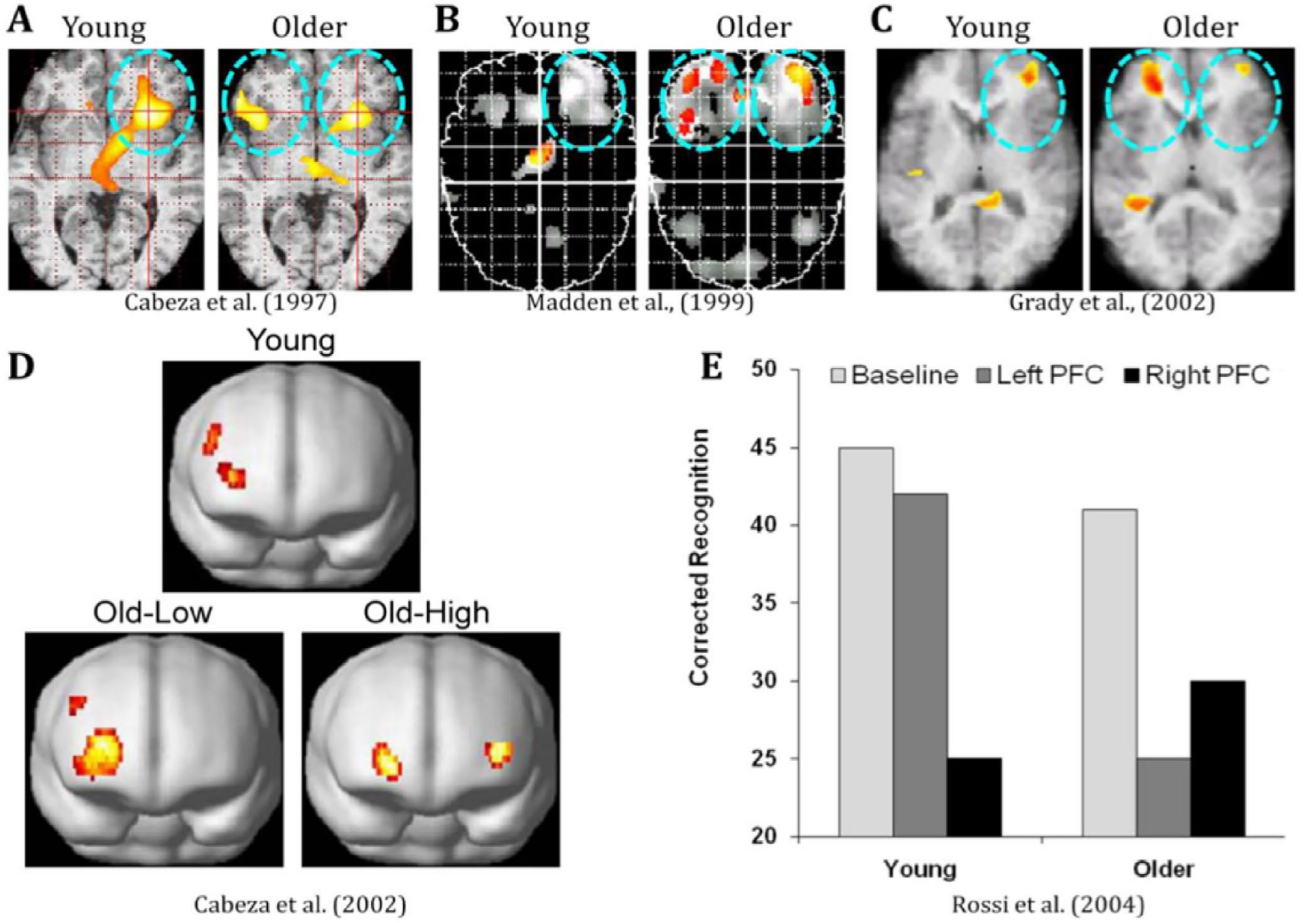
Findings of functional neuroimaging studies showed that prefrontal activity during episodic memory retrieval was bilateral in older adults and children, but lateralized in younger adults, forming an inverted U. (A) Following Cabeza et al. (1997); Madden et al. (1999); (C) Grady et al. (2002); (D) Cabeza (2002); (E) hexagonal interaction of age groups and rTMS effects during retrieval (adapted from Rossi et al. 2004).

Interestingly, Figure 10 supports the notion that aging is linked to both a rise in the FC of homologous regions in the two hemispheres and a more fMRI bilateral activation pattern (Davis et al. 2012). Daselaar et al.'s (2006) fMRI study examined how aging affected activity during a word recognition test. Functional connectivity was assessed by assessing fluctuations in activity across trials. In association with successful retrieval, the mediotemporal region (MTL) (hippocampus) and posterior cortical regions (parietal and retrosplenial cortices) are less connected in older people, although the entorhinal cortex, another MTL region, and bilateral dorsolateral PFC regions are significantly more connected (Figure 10A). Age‐related shifts are also noted from stronger FC with posterior regions in young adults to stronger connectivity with PFC regions in older adults. While encoding neutral and emotional images, St. Jacques et al. (2009) examined individuals of all ages. The amygdala was the main focus of connection study because of the emotional character of the stimuli. Once again, FC was evaluated both within and between trials. Although the amygdala was equally engaged in both age groups during successful emotional encoding, young adults' amygdala connectivity was stronger with the hippocampus, a posterior region, while older adults' amygdala connectivity was stronger with bilateral dorsolateral PFC regions (Figure 10B). We can hypothesize that the age‐related increase in PFC connectivity in this and the Daselaar et al. (2006) study represents a strategic shift in memory processing, with school‐aged children acting similarly to the older adults and older adults using more top‐down control mechanisms when performing successful memory tasks (Açık et al. 2010), possibly to compensate for the MTL's diminished functioning. This then produces an inverted U function. Additionally, functional neuroimaging studies, represented in Figure 11, showed that prefrontal activity during episodic memory retrieval was bilateral in older adults and children, but lateralized in younger adults, forming an inverted U.

The relationship between brain function and behavior also varies across the lifespan. For instance, the period between ages 22 and 34 is often considered a peak phase for cognitive functioning, serving as a reference point for comparing brain‐behavior relationships in other age groups (Petrican et al. 2017). This peak period is characterized by optimal neural efficiency and cognitive performance, which gradually decline with age.

Studies have shown that different brain networks exhibit distinct developmental trajectories. Throughout life, the DAN, which includes areas like the superior parietal lobe and frontal eye field, displays comparatively constant levels of activity. According to Li et al. (2024), the frontoparietal control network, exhibits an inverted U‐shaped trajectory, peaking in activity during young adulthood and declining as people age. This dissociation highlights the hierarchical effects of aging on brain function, where some networks remain stable while others deteriorate.

Moreover, cognitive variables and abilities are influenced by age in both children and adults. However, the nature of these influences can differ across age groups. For example, crystallized abilities, which involve the use of knowledge and experience, show a significant relationship with age in children, while working memory is more affected in adults (Salthouse and Davis 2006). This indicates that different cognitive functions may be more or less vulnerable to age‐related changes depending on the stage of life.

Inhibition, or the ability to suppress irrelevant information and responses, plays a crucial role in cognitive performance and is particularly important in older adulthood. Research suggests that age‐related declines in cognitive performance are often linked to reductions in inhibitory control (Zimmermann and Meier 2006). This decline in inhibition can affect various cognitive tasks, leading to slower processing speeds and increased susceptibility to distractions.

Furthermore, the concept of the “inverted U” relationship between movement and cognition is evident in adult cognitive development. High levels of physical activity are associated with better cognitive function, but this relationship can vary depending on the intensity and type of activity. For instance, moderate physical activity may provide optimal benefits for cognitive health, while excessive or insufficient activity may not yield the same positive effects (Morse et al. 2011).

### 5.9 Cognitive Decline in Aging

Aging and decline in cognitive function are complex processes influenced by a myriad of factors, including environmental exposures, lifestyle choices, and genetic predisposition. As individuals age, there is a notable decline in various cognitive domains, which can be attributed to both functional and changes in the brain. Cognitive abilities such as EF, memory, and processing speed tend to decline with age (Ferguson et al. 2021; Zimmerman et al. 2021). This decline is not uniform across all cognitive domains, and different cognitive functions may exhibit distinct patterns of change over the lifespan.

**FIGURE 12:**
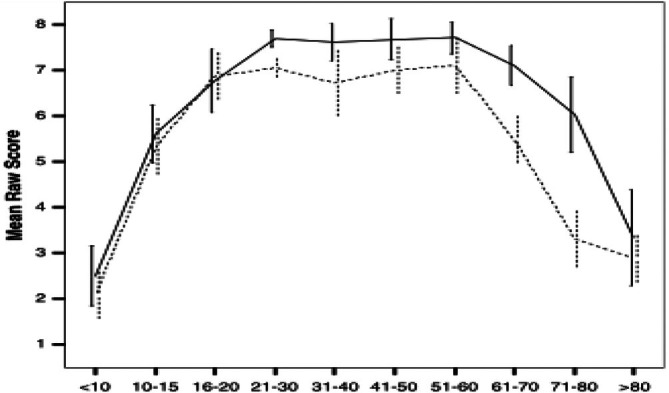
Findings from a sizable cross‐sectional (*N* = 1704) ranging in age from 4 to 95. An examination of the impact of individuals' age on their performance on eight Piagetian water‐level activities showed that the capacity to correctly complete the tasks increased with age in children and adolescents, peaked in middle‐aged participants, and decreased in older people.

Among the neuromotor and neurocognitive functions that reportedly are evident in a significant proportion of the elderly are, as we had earlier indicated, the return of primitive or primary reflexes, also referred to as frontal release signs, and associated cognitive decline, thereby linking these systems (Leisman et al. 2022, 2023). With the return of frontal release, signs, cognitive effects may be noted, as evidenced in Figure 12.

One significant aspect of cognitive aging is the decline in network segregation, which refers to the brain's ability to maintain distinct functional networks (Pedersen et al. 2021). This decline is a key feature of typical development from childhood to adulthood and continues into old age (Petrican et al. 2017). Reduced network segregation can lead to less efficient cognitive processing and is associated with various age‐related cognitive deficits.

Moreover, the interplay between genetic factors and cognitive reserve plays a significant role in cognitive aging. Intellectual and psychosocial factors, such as engaging in mentally stimulating activities, can enhance cognitive reserve and potentially mitigate the effects of brain pathologies.

White matter integrity is another critical factor in cognitive aging. Age‐related degradation in white matter is not solely due to cognitive dysfunction or increased dementia risk. Instead, it reflects a broader pattern of brain aging, with greater effects observed in posterior regions compared to younger‐old adults (Groh and Simons 2025). This white matter anterior‐to‐posterior gradient in aging underscores the complexity of brain changes that occur with advancing age.

Additionally, the volume and morphometry of brain structures, particularly the hippocampus, exhibit significant changes in the oldest old adults. These changes are indicative of a more widespread vulnerability in advanced age, which may be exacerbated by factors such as the APOE genotype and amyloid‐beta pathology (Merenstein and Bennett 2022).

One of the key findings in the study of cognitive aging is the presence of an inverted U‐shaped trajectory in certain cognitive functions. Based on this pattern, cognitive capacities peak in early adulthood, then deteriorate in later years after improving during childhood and youth. For instance, EF, which includes tasks such as planning, problem‐solving, and inhibitory control, shows a developmental improvement from adolescence to young adulthood, followed by a decline throughout adulthood (Ferguson et al. 2021). This pattern is consistent with the notion that cognitive control regions in the brain, such as the frontoparietal network, exhibit an inverted U‐shaped trajectory.

Changes in the structure and function of the brain are one of the many reasons why cognitive abilities deteriorate with age. Cognitive performance deficits have been associated with age‐related alterations in the brain, including decreases in white matter integrity (Schilling et al. 2023) and gray matter volume (Christova and Georgopoulos 2023). For example, the DAN (Lee et al. 2023; Droby et al. 2022), which is involved in attentional orientation, shows a relatively flat pattern of change with age, whereas the frontoparietal control network, which underlies cognitive control, exhibits a more pronounced decline (Wei et al. 2022). White matter hyperintensities (WMH) and microstructural changes in the brain are also significant factors in cognitive performance among adults (Wiseman et al. 2018; Li et al. 2021). These white matter measures, however, are unable to distinguish across cognitive status subgroups in the oldest‐old persons, indicating that other factors might be more important in causing cognitive failure in later life. These hierarchical age effects on brain function highlight the complexity of cognitive aging and the need to consider multiple brain regions and networks.

In adulthood, cognitive flexibility, which encompasses the ability to switch between tasks and maintain multiple task sets, shows distinct patterns. While switching costs do not exhibit significant age‐related changes, mixing costs tend to increase with age. This indicates that maintaining multiple task sets becomes more challenging as individuals age (Ferguson et al. 2021).

Negative stereotypes about aging often include assumptions of universal cognitive decline. However, this is not entirely accurate, as cognitive performance can vary widely among individuals. Some older adults maintain high levels of cognitive function, potentially due to factors such as cognitive reserve and lifestyle choices (Schaie 2010).

Functional connectivity within the brain also changes with age. A decline in network segregation, which is the ability of different brain regions to function independently, is a key aspect of aging. This reduced segregation is associated with decreased cognitive performance, highlighting the importance of maintaining FC for cognitive health (Petrican et al. 2017).

Moreover, longitudinal studies have shown that changes in whole‐brain FC patterns are related to global cognitive decline in older adults. These findings underscore the importance of monitoring and potentially intervening in FC to support cognitive health in aging populations (Merenstein and Bennett 2022).

To mitigate the impact of brain deterioration on cognitive performance, cognitive reserve is essential. Despite having comparable degrees of brain disease, people with stronger cognitive reserve may do better cognitively than those with lower cognitive reserve. According to Wang et al. (2019), this implies that cognitive reserve can act as a buffer against the negative consequences of brain aging and disease.

An important issue to understand is the connection between physical activity and cognitive deterioration. It has been demonstrated that physical activity improves cognitive performance, which may help to offset some of the age‐related decreases. The relationship between physical exercise and cognitive performance emphasizes how crucial it is to lead a balanced lifestyle to support older individuals' general well‐being (Sheffler et al. 2022).

In addition to structural changes in the brain, cognitive decline in aging is also influenced by changes in cognitive processing strategies. Older adults may rely more on automatic processing and less on controlled processing, which can affect their performance on tasks that require executive control. The idea of age‐related changes in cognitive control mechanisms is further supported by studies that have shown reductions in executive functioning across the adult years using paradigms like the Wisconsin Card Sorting Task (Craik and Bialystok 2006).

Furthermore, the variability in cognitive decline among individuals suggests that other factors, such as genetics, education, and lifestyle, play a role in shaping cognitive trajectories (Erickson et al. 2022). For instance, some individuals may experience significant declines in certain cognitive abilities, while others maintain relatively stable performance. This variability highlights the need for personalized approaches to understanding and addressing cognitive aging (Pauls et al. 2013). To treat age‐related declines and progression into neurodegenerative illnesses, it may benefit the late‐life aging population to employ “neuroprotective” lifestyles such as staying intellectually engaged, maintaining cardiovascular activity, minimizing chronic stressors, and growing accustomed to a brain‐healthy diet. In relevance, it has been suggested that MCI, a transitional stage between normal aging and dementia associated with poor performance on memory tasks and subjective memory complaints, could be utilized to explore biomarkers reflecting prodromal stages of AD. In response, we suggest that particular electroencephalography measures (i.e., event‐related oscillations and resting EEG) associated with MCI could provide reliable early biomarkers to differentiate healthy controls from premorbid neuropsychiatric populations, including MCI patients and prodromal dementia patients (Deiber et al. 2009; Finnigan and Robertson 2011), allowing the implementation of early non‐invasive oscillatory electrophysiological interventions (Thut et al. 2011) that could potentially postpone cognitive decline and preserve executive cognitive functions in older healthy people.

#### 5.9.1 Aging and Workplace Cognitive Demands

Workplace cognitive demands are a critical aspect of adult cognitive functions, influencing both productivity and overall well‐being. The cognitive requirements in the workplace can vary significantly depending on the nature of the job, the individual's role, and the specific tasks they are required to perform. These demands often encompass a range of cognitive abilities, including EF, memory, attention, and problem‐solving skills. EFs play a crucial role in workplace settings. These functions include planning, decision‐making, conflict resolution, and the ability to manage multiple tasks simultaneously. For instance, individuals with extensive frontal lobe lesions may exhibit automatic behaviors dominated by the current context, such as responding to the sight of sewing materials by sewing or eating when presented with food, indicating a loss of control over their actions (Craik and Bialystok 2006). This highlights the importance of intact EF for effective workplace performance.

The relationship between cognitive abilities and workplace performance is further complicated by age‐related changes. Research indicates that cognitive abilities such as word fluency, number, and spatial orientation peak at different ages, with some abilities not showing significant decline until the late 60s (Schaie 2010). This suggests that older adults may still maintain high levels of cognitive function in certain areas, which can be beneficial in the workplace. However, the decline in other cognitive abilities may necessitate adjustments in job roles or the implementation of supportive measures to maintain productivity.

The brain maintenance theory suggests that preserved cognitive functioning in older adults is associated with minimal changes in brain aging markers (Habeck et al. 2017; Nilsson and Lövdén 2018). Studies indicate that older individuals who maintain cognitive function for a decade or longer tend to have higher volumes in regions such as the entorhinal cortex, parahippocampal gyrus, and hippocampus (Devanand et al. 2007; Köhncke et al. 2021).

Beyond age‐related changes, an individual's cognitive reserve, including larger brain volumes, also influences their ability to meet cognitive demands in the workplace (Habeck et al. 2017; Nilsson and Lövdén 2018). According to the brain reserve hypothesis, individuals with higher cognitive reserve are more likely to retain normal cognitive function despite brain pathology, providing an advantage in coping with cognitive challenges even in later life.

## 6 Discussion

Brain development across the lifespan exhibits an inverted U‐shaped trajectory, indicating that certain aspects of brain connectivity and cognitive abilities peak at middle age before declining in older adulthood, and for various reasons resemble early childhood.

Structural connectivity strength in the brain follows an inverted U‐shaped trajectory, peaking in the early 30s. This pattern is observed across all brain regions, including hub regions that are central to brain network organization. While connectivity strength generally declines with age, the organization of these hub regions into a rich club remains stable and even becomes more pronounced, suggesting a resilient structure that supports cognitive functions even in aging. Thatcher (2009), in reviewing his work over many years, noted that brain development across the lifespan follows an inverted “U‐shaped model,” where optimal complexity is achieved at the apex of differentiation (specialization of functions) and integration (coordination of functions). The maturation of cognitive abilities occurs in growth spurts, represented as a trajectory on this “inverted U‐shaped” model, indicating periods of increased efficiency and complexity. Developmental processes include cycles of synaptic overproduction followed by pruning, reflecting a hierarchical organization that contributes to the inverted “U”‐shaped function of complexity. The maturation of cognitive abilities also aligns with this inverted U model. Growth spurts in cognitive development manifest as peaks in complexity and efficiency during certain life stages, particularly from birth to around 16 years of age. This reflects a balance between integration and differentiation of cognitive processes, suggesting that optimal cognitive functioning occurs at this apex of development.

In the context of intrinsic FC related to social cognition, different networks exhibit distinct developmental trajectories. For instance, the theory of mind (ToM) network follows a pronounced inverted U‐shaped trajectory, indicating that its connectivity increases to a peak before declining. This suggests that the ability to engage in complex social reasoning may also peak at a certain age before experiencing a decrease.

The underlying mechanisms driving this inverted U‐shaped trajectory are influenced by various factors, including synaptic development characterized by cycles of overproduction and pruning of synapses. These cycles contribute to the hierarchical organization and efficiency of brain networks, which are essential for cognitive function throughout life. Overall, the inverted U‐shaped model of brain development highlights critical phases where both structural and functional aspects of the brain reach their peak before experiencing decline. This model underscores the importance of understanding how brain networks adapt and change across the lifespan, particularly to cognitive abilities and resilience in aging.

Many of the brain network organizational principles are set before birth, continuing postpartum with significant developmental alterations. These consist of densely connected, rich, and topologically central hub areas. Comparatively little is known about subsequent development across the lifespan, even though numerous studies have charted the developmental trajectories of brain connection and brain network organization during childhood and adolescence. Riedel et al. (2022) conducted a cross‐sectional study on the evolution of the structural brain network in 8066 people ranging in age from 5 to 80. An “inverted‐U”‐shaped trajectory with a vertex in the early 30s was seen in the strength of structural connections across all brain areas. The hub regions' connectivity strength followed a similar pattern, and their identities were consistent across all age groups.

We know that connectivity strength decreases with age; the arrangement into a rich club not only remains the same but also becomes more noticeable, most likely as a result of key connections being selectively spared from age‐related connectivity loss. A “first come, last served” model of neurodevelopment, in which the first principles to emerge are the last to deteriorate with age, is consistent with the stability of rich club organization in the face of general age‐related decline. Rich club organization is very good for higher cognition and communication. Thus, a robust, rich club may serve as a neural reserve to maintain cognitive ability in the aging brain and protect against functional loss in late adulthood.

**FIGURE 13:**
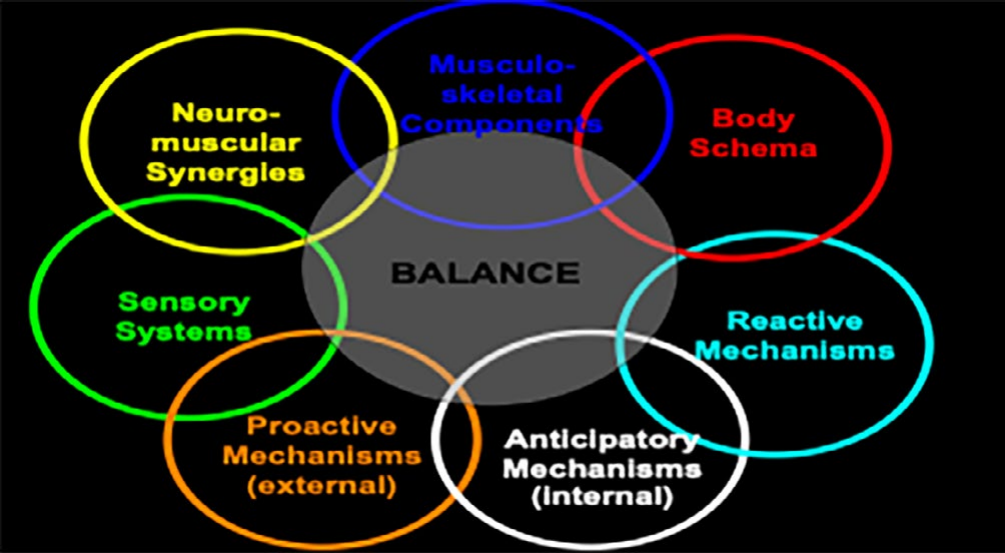
Each of the components of integrated or balanced function changes dynamically throughout the lifespan of the individual, whose components, when compared between early childhood and aged individuals, will resemble an inverted U.

If one were to take all throughout the lifespan as represented in Figure 13, one would be attempting to achieve a homeostasis of the components of development, ostatic balance with varying degrees of success, with an unchanging balance achieved upon death. In the interim, neuromuscular synergies are similar in childhood and among the aged but significantly different than those in neurotypical adults (Shafizadeh et al. 2019; Monaco et al. 2010). Sensory systems are likewise developing and integrating, growing from small‐world networks in childhood to more global networks in adulthood and then reverting again to more modular functioning in advanced aging (Edde et al. 2021). While proactive response mechanisms do not significantly change over one's lifetime (Smittenaar et al. 2015), cognitive‐motor anticipatory mechanisms have been reported to demonstrate a significant decline (Stöckel et al. 2017). Manard et al. (2017) have observed that aging affects neural networks associated with reactive and proactive cognitive control differentially. These age‐related changes are similar to those observed in young adults with low dopamine availability, suggesting that a general mechanism (prefrontal dopamine availability) may modulate brain networks associated with various kinds of cognitive control and render the aged individual in a state of functionality closer to that of early childhood, an inverted U‐type relationship.

The concept of the “inverted U” relationship between movement and cognition throughout the human lifespan underscores the intricate interplay between physical activity and cognitive function. This relationship suggests that an optimal level of physical activity maximizes cognitive function, while insufficient activity may lead to suboptimal cognitive outcomes. This phenomenon is observed across various stages of life, from fetal development to old age, highlighting the importance of maintaining an appropriate level of physical activity to promote overall well‐being.

During fetal development, the foundation for future cognitive and physical health is established. As children grow, physical activity continues to play a crucial role in cognitive development. Engaging in regular physical activity has been shown to enhance various aspects of cognitive function, including attention, memory, and EF. These cognitive benefits are particularly important during the school years when academic performance and learning are closely linked to cognitive abilities.

In adulthood, the relationship between physical activity and cognition remains significant. Regular physical activity is associated with a reduced risk of cognitive decline and neurodegenerative diseases such as Alzheimer's disease. In older adults, physical activity continues to be a key factor in preserving cognitive function. The “inverted U” relationship between movement and cognition suggests that there is an optimal level of physical activity that maximizes cognitive function. Both insufficient and excessive physical activity can lead to suboptimal cognitive outcomes, indicating the need for a balanced approach to physical activity. This balance, as reflected in Figure 9, is crucial at all stages of life, from fetal development to old age, to promote overall well‐being and cognitive health.

Thatcher (2009), in summarizing his work over many years, noted that brain development across the lifespan follows an inverted “U”‐shaped model, where optimal complexity is achieved at the apex of differentiation (specialization of functions) and integration (coordination of functions). The maturation of cognitive abilities occurs in growth spurts, represented as a trajectory on this inverted U‐shaped model, indicating periods of increased efficiency and complexity. Developmental processes include cycles of synaptic overproduction followed by pruning, reflecting a hierarchical organization that contributes to the inverted “U”‐shaped function of complexity.

Fan and Yan (2022) also noted that the theory of mind (ToM) network exhibits a pronounced inverted U‐shaped trajectory, indicating that its connectivity increases to a peak and then decreases with age. The empathy network shows a quadric‐concave trajectory, suggesting that its connectivity. He et al. (2022) additionally noted that the frontoparietal and default mode networks exhibit an inverted U‐shape contribution pattern from developmental stages to aging stages, indicating varying levels of involvement across the lifespan. Different brain regions, particularly in the frontal lobe, play crucial roles in age prediction during both developmental and aging phases, with the thalamus showing stability throughout.

While the “inverted U” model provides a useful framework for understanding general trends in brain development, the development, and decline of cognitive performance across the lifespan, it does not capture all of the exceptions suggested by the model. For example, individuals with neurodegenerative diseases or neurological impairments often exhibit trajectories that differ significantly from the classic inverted U shape. Furthermore, there are cases where cognitive function can coexist with severe physical decline, suggesting that cognitive resilience is not always directly dependent on physical activity and can also exist in other circumstances.

In addition, the initiation of normal physical activity later in life may offer limited correction for irreversible neurodevelopmental deficits resulting from inadequate early motor experiences, highlighting the critical role of physical engagement early in life in shaping brain architecture. Environmental, genetic, and pathological factors further modulate cognitive pathways, complicating the predictive power of simple models. Thus, while the inverted U provides an important heuristic, it must be placed in a broader context that considers individual variation, compensatory mechanisms, and multifactorial influences on brain health across the lifespan.

## 7 Conclusion

The exploration of the interconnectedness between movement and cognitive function across the lifespan reveals a complex and dynamic relationship. The concept of the “inverted U” trajectory underscores the importance of maintaining an optimal level of physical activity to maximize cognitive function. This relationship is evident from fetal development through old age, highlighting the necessity of a balanced approach to physical activity for overall well‐being. During fetal development, maternal physical activity has a positive influence on fetal brain development, laying the foundation for future cognitive and physical health. As children grow, engaging in regular physical activity enhances various aspects of cognitive function, including attention, memory, and EF. These benefits are particularly crucial during the school years, when academic performance is closely linked to cognitive abilities. In adulthood, regular physical activity continues to play a significant role in preserving cognitive function and reducing the risk of neurodegenerative diseases such as Alzheimer's disease. The “inverted U” relationship suggests that both insufficient and excessive physical activity can lead to suboptimal cognitive outcomes, emphasizing the need for a balanced approach. In older adults, physical activity remains a key factor in maintaining cognitive health. Regular exercise can slow the progression of cognitive decline and improve the quality of life. The decline in network integrity observed in older age highlights the importance of integrating physical and cognitive activities to promote overall well‐being.

These findings highlight the fundamental and continuous role of motor activity in shaping cognitive function, from early brain development through to neurodegeneration in aging, emphasizing the importance of integrating motor‐cognitive perspectives across the lifespan in both research and practice.

## Author Contributions

**Gerry Leisman:** conceptualization (lead), formal analysis (lead), investigation (lead), project administration (lead), resources (lead), supervision (lead), validation (supporting), visualization (lead), writing – original draft (lead), writing – review and editing (lead). **Rahela Alfasi:** data curation (supporting), investigation (supporting), validation (supporting), visualization (lead), writing – original draft (supporting), writing – review and editing (supporting). **Oded Meiron:** investigation (supporting), methodology (supporting), writing – original draft (supporting), writing – review and editing (supporting). **Amedeo D'Angiulli:** conceptualization (equal), project administration (supporting), supervision (lead), writing – original draft (equal), writing – review and editing (equal).

## Funding

The authors have nothing to report.

## Conflicts of Interest

The authors declare no conflicts of interest.

## Related WIREs Articles

Early experience and brain development

Educating executive function

## Data Availability

Data sharing is not applicable to this article as no new data were created or analyzed in this study.
